# Identification and Classification of Conserved RNA Secondary Structures in the Human Genome

**DOI:** 10.1371/journal.pcbi.0020033

**Published:** 2006-04-21

**Authors:** Jakob Skou Pedersen, Gill Bejerano, Adam Siepel, Kate Rosenbloom, Kerstin Lindblad-Toh, Eric S Lander, Jim Kent, Webb Miller, David Haussler

**Affiliations:** 1 Center for Biomolecular Science and Engineering, University of California Santa Cruz, Santa Cruz, California, United States of America; 2 Broad Institute of MIT and Harvard, Cambridge, Massachusetts, United States of America; 3 Center for Comparative Genomics and Bioinformatics, Pennsylvania State University, University Park, Pennsylvania, United States of America; 4 Howard Hughes Medical Institute, University of California Santa Cruz, Santa Cruz, California, United States of America; Sanger Institute, United Kingdom

## Abstract

The discoveries of microRNAs and riboswitches, among others, have shown functional RNAs to be biologically more important and genomically more prevalent than previously anticipated. We have developed a general comparative genomics method based on phylogenetic stochastic context-free grammars for identifying functional RNAs encoded in the human genome and used it to survey an eight-way genome-wide alignment of the human, chimpanzee, mouse, rat, dog, chicken, zebra-fish, and puffer-fish genomes for deeply conserved functional RNAs. At a loose threshold for acceptance, this search resulted in a set of 48,479 candidate RNA structures. This screen finds a large number of known functional RNAs, including 195 miRNAs, 62 histone 3′UTR stem loops, and various types of known genetic recoding elements. Among the highest-scoring new predictions are 169 new miRNA candidates, as well as new candidate selenocysteine insertion sites, RNA editing hairpins, RNAs involved in transcript auto regulation, and many folds that form singletons or small functional RNA families of completely unknown function. While the rate of false positives in the overall set is difficult to estimate and is likely to be substantial, the results nevertheless provide evidence for many new human functional RNAs and present specific predictions to facilitate their further characterization.

## Synopsis

Structurally functional RNA is a versatile component of the cell that comprises both independent molecules and regulatory elements of mRNA transcripts. The many recent discoveries of functional RNAs, most notably miRNAs, suggests that many more are yet to be found. Computational identification of functional RNAs has traditionally been hampered by the lack of strong sequence signals. However, structural conservation over long evolutionary times creates a characteristic substitution pattern, which can be exploited with the advent of comparative genomics. The authors have devised a method for identification of functional RNA structures based on phylogenetic analysis of multiple alignments. This method has been used to screen the regions of the human genome that are under strong selective constraints. The result is a set of 48,479 candidate RNA structures. For some classes of known functional RNAs, such as miRNAs and histone 3′UTR stem loops, this set includes nearly all deeply conserved members. The initial large candidate set has been partitioned by size, shape, and genomic location and ranked by score to produce specific lists of top candidates for miRNAs, selenocysteine insertion sites, RNA editing hairpins, and RNAs involved in transcript auto regulation.

## Introduction

Many new classes of functional RNA structures (fRNAs), such as snoRNAs, miRNAs, splicing factors, and riboswitches [1–3], have been discovered over the last few years. These structures function both as independent molecules and as part of mRNA transcripts. These recent discoveries verify that fRNAs fulfill many important regulatory, structural, and catalytic roles in the cell, and suggest that perhaps only a small fraction of these fRNAs are currently identified [1,3,4].

The development of computational methods that can efficiently identify fRNAs by comparative genomics has been hampered by the fact that fRNAs often exhibit only weakly conserved primary-sequence signals [5]. Fortunately, the stem-pairing regions of fRNA structures evolve mostly with a characteristic substitution pattern such that only substitutions that maintain the pairing capability between paired bases will be allowed. This leads to compensatory double substitutions (e.g., GC ↔ AU) and to a few types of compatible single substitutions (e.g., GC ↔ GU); the latter made possible by RNA's ability to form a non–Watson-Crick pair between G and U. This evolutionary signal can be exploited for comparative identification of fRNAs [6–12].

The many non-human vertebrate genomes now sequenced can be aligned against the human genome, leading to a multiple alignment with considerable information about the evolutionary process at every position [13–15]. Given a diverse enough set of genomes, comparative methods that can make effective use of this evolutionary information should in principle be able to efficiently identify the conserved human fRNAs. We have developed a comparative method called EvoFold for functional RNA-structure identification in multiple sequence alignments. EvoFold makes use of a recently devised model construction, a phylogenetic stochastic context-free grammar (phylo-SCFG) [10,16,17], which is a combined probabilistic model of RNA secondary structure and sequence evolution. Phylo-SCFGs use stochastic context-free grammars (SCFGs) [18,19] to define a prior distribution over possible RNA secondary structures, and a set of phylogenetic models [20–22] to evaluate how well the substitution pattern of each alignment column conforms with its secondary-structure annotation. EvoFold uses a very general model of RNA secondary structures that allows it to model everything from short hairpins to complex multiforking structures, including novel structures not seen in its training set. The substitution process explicitly models co-evolution of paired bases within the structure using the phylogenetic tree and evolutionary branch lengths relating the sequences of the alignment. Stem-pairing regions are detected not only by the presence of compensatory substitutions, but also by the presence of compatible single substitutions and the overall slower rate of evolution. We have built a human-referenced eight-way vertebrate whole-genome alignment and used EvoFold to search for functional RNAs in the human genome. This search resulted in a total of 48,479 candidate RNA structures. Based on estimates of the false-positive rate, which unfortunately are associated with very large uncertainties, we estimate that the candidate set contains approximately 18,500 substructures of approximately 10,000 RNA transcripts. These numbers are derived using an estimated false-positive rate of 62%. Among the highest-scoring candidates, where the estimated false-positive rate is much lower, this screen finds a large number of known functional RNAs, and contains new candidate miRNAs, selenocysteine insertion sites, RNA editing hairpins, RNAs involved in transcript auto regulation, and many folds that form singletons or small functional RNA families of completely unknown function.

## Results

**Figure 1:**
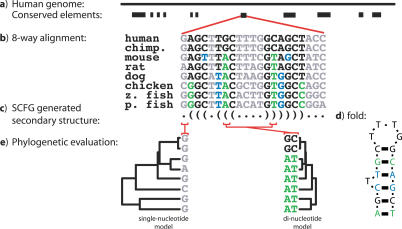
Outline of EvoFold Prediction Method (A) Schematic representation of human genome and conserved elements. The conserved elements define the input alignments. (B) Segment of eight-way genomic alignment. (C) The SCFG of the fRNA model defines a distribution over all possible secondary-structure annotations. One of the many possible secondary structures is shown in parenthesis format. Substitutions in pairing regions of the alignment are color-coded relative to human: compensatory double substitutions are green, and compatible single substitutions are blue. (D) Color-coded fold corresponding to the secondary-structure annotation of the alignment. (E) Two phylogenetic models are used to evaluate the possible secondary-structure annotations: unpaired columns are evaluated using a single-nucleotide phylogenetic model. Paired columns are combined and evaluated using a di-nucleotide phylogenetic model. Horizontal branch lengths reflect the expected number of substitutions.

We constructed a whole-genome alignment of the human [23], chimpanzee [24], mouse [25], rat [26], dog, chicken [27], zebra fish, and puffer fish [28] genomes using the MULTIZ program [13,29]. From this alignment we assembled a set of human genome segments where at least four other species are aligned and the pattern of substitution shows evidence of negative selection using the PhastCons method [15]. These segments were further filtered to remove retroposed genes, simple/low-complexity repeats, segments with mitochondrial chromosome homology, and segments that were not clearly in the orthologous locations with respect to neighboring genes in both the human and mouse genomes (“nonsyntenic human-mouse matches”). The resulting set defines 1,181,107 conserved segments spanning 3.7% of the reference human genome. We applied the EvoFold algorithm, illustrated in Figure 1, to each of these conserved segments. This resulted in a total of 48,479 candidate RNA folds with more than five pairing bases that span 0.07 % of the human genome at the base level (see Figure S1 for length distribution). These can be interactively explored or retrieved in bulk from the University of California Santa Cruz (UCSC) Genome Browser (http://genome.ucsc.edu, Protocol S1).

We classified these candidate folds according to three different criteria: their size, their genomic location, and their overall shape. We distinguished two size ranges: short (between five and 15 pairing bases, 39,075 folds) and long (more than 15 pairing bases, 9,404 folds); five types of genomic location: coding (12,736 folds), 3′UTR (3,331 folds), 5′UTR (334 folds), intronic (11,777 folds), and intergenic (20,301 folds); and four shape-types: hairpins (42,964 folds),Y-shaped (3,479 folds), clover-shaped (250 folds), and more complex shapes (1,786 folds). This scheme results in 40 different RNA fold prediction categories. Candidate folds were also clustered by proximity in the genome or overlap with cDNAs into sets of folds that are likely to be part of a single underlying RNA transcript. This grouped the 48,479 candidate RNA folds into 23,287 candidate structure–containing transcripts. Finally, the folds within each category were ranked by a length-normalized likelihood-ratio score that we call the folding potential score (fps), and a shuffling scheme was used to tentatively estimate the rate of false-positive predictions in each category as a function of score (Materials and Methods, Figures S2 and S3, Tables S1 and S2).

We mapped all available human and non-human mRNAs and ESTs to the human genome and determined the enrichment of hits to our set of candidate RNA folds relative to the background hit rate in genomic DNA. These were found to vary from 3.6× (cDNA from humans) to 11.4× (non-human EST). This is significantly higher than the enrichments observed for the full set of conserved elements from which these candidates were chosen (Figure S4).

We also found that predictions at known fRNAs generally score higher on the strand of the fRNA compared to its reverse complement (this is, e.g., the case for 89% of the known miRNAs we predict). The asymmetry is primarily caused by the ability of GU (or UG) to pair, but not its reverse complement AC (CA). Since the most common types of substitutions in RNA stems involve GU (or UG) pairs, this can have a pronounced effect on the EvoFold score, thus allowing the strand association of a fold to be inferred by comparing the score of an alignment with the score of its reverse complement. In cases where the candidate RNA is contained in a known transcript, the EvoFold score for the sense strand (i.e., the strand complementary to the template strand for transcription) is often significantly higher than for the anti-sense strand (Table S3). Because this is similar to the effect observed for known fRNAs, this provides circumstantial evidence that many of these predictions are new fRNAs. However, part of this effect may be due to compositional asymmetries, possibly due to transcription-mediated repair [30], or the influence of other sense-strand associated functional elements (see Protocol S1).

**Figure 2:**
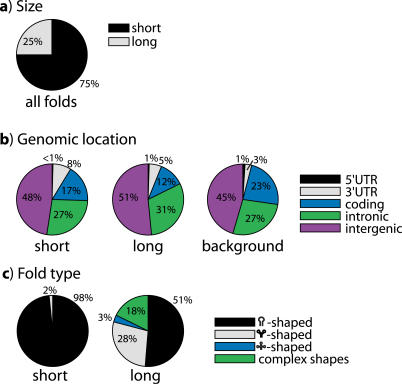
Breakdown of Types of RNA Folds Detected in the Human Genome Based on True Positive Estimates See Materials and Methods, Validation section. Folds are classified according to (A) size (number of pairing bases), (B) location in the genome, and (C) shape. The relative abundance of each class of folds is indicated. For (B), also shown is the genomic span of the conserved segments relative to their genomic location, for comparison.

Using a shuffling approach, we estimate that the set of 48,479 candidates contain 18,500 partially correct fRNAs (see Materials and Methods, Validation section). However, this estimate is associated with huge uncertainties inherent to the shuffling approach and should only be viewed as a first approximation based on the available data (see Discussion). Based on the shuffling approach and the genomic distribution of the candidates, we estimate, conditional on the above-mentioned uncertainties, that our predictions comprise about 10,000 human RNA transcripts: 2,200 of which are transcripts of protein-coding genes that harbor functional RNAs in their UTRs or overlapping their coding region, and the remainder being fRNA genes. After correcting for the shuffling-based estimates of false-positive rates, the folds break down into the different sizes, locations, and shapes as shown in Figure 2.

Three quarters of the predicted folds are short. These are likely to represent a mix of small complete folding units and partial predictions of larger folds, where only a small core element had sufficient evolutionary covariation to be detected by our method. Among the long folds, about 82% are intergenic or intronic, 5.5% are in 3′UTRs, 0.5% in 5′UTRs, and a surprising 12% (550 folds) overlap known coding regions. These are discussed further below. As expected, the small folds are predominantly single hairpins; there are usually not enough paired bases in these to support more complex stable structures. The long folds show a more varied shape distribution, but are also dominated by simple hairpins. Again, since these are often partial structural predictions, this breakdown is likely to be somewhat biased toward the simpler fold types.

Because EvoFold is designed to look for RNAs that are conserved in structure and remain in the same genomic context in all vertebrates, there are likely to be additional fRNAs not detected in this survey. There are some classes of known functional RNAs that are too mobile or rapidly evolving for EvoFold to detect, such as tRNAs and snoRNAs. The vertebrate tRNAs spawn many lineage-specific copies that land in different places in the genome, most of which are pseudogenes, so that the remaining functional copies often end up in a different genomic context in different vertebrate lineages [27]. As a result, more than 99% of the functional human tRNAs fail the filter we applied that removes nonsyntenic matches between human and mouse, and hence are absent in our set of predicted folds. In contrast, most snoRNAs are missing from our set of predicted folds either because they have too few base pairs (bp), e.g., 4–5 bp in the CD-box snoRNAs, or have experienced too many structural changes in vertebrate evolution. We observe that 32% of the bp of known deeply conserved snoRNAs could not be formed in fish or chicken, causing a conflict with the overall structural signal EvoFold is designed to detect. The signal recognition particle RNA and the Y RNAs are also missed due to their evolutionary mobility. On the other hand, RNase P RNA and both the U11 and U12 spliceosomal RNAs are well conserved and detected by this screen. Based on our current methods, we cannot predict how many more, as-yet-undiscovered, classes of highly mobile or rapidly evolving RNAs there are in vertebrate genomes.

For other known classes of RNAs, such as miRNAs, EvoFold achieves a high rate of sensitivity, finding nearly all known members. To evaluate EvoFold's sensitivity, we performed a 5-fold cross-validation test using various curated sets of known RNAs. These tests showed that EvoFold is quite good at detecting some known classes of RNAs, such as miRNAs and Histone 3′UTR stem loops (Table 1). Despite the fact that Histone 3′UTR stem loops have stems containing only 6 bp, they are predicted very accurately: 97% predicted with 100% correct structure.

**Table 1:**
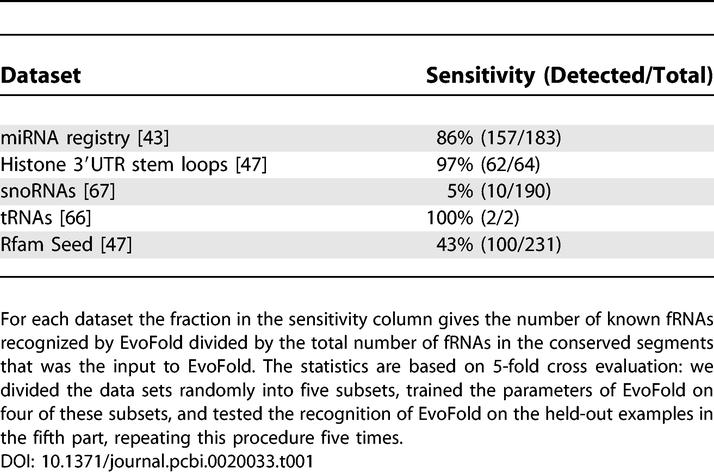
EvoFold Sensitivity

Since the fps used by EvoFold ranks deeply conserved compact folds highly, we also defined an alternative score directly based on the substitution evidence and used it to define a ranked set of 517 ncRNA candidates (see Protocol S1). This score, for example, top-ranks the U11 and U12 spliceosomal RNAs mentioned above. The second-highest ranked clover-shaped fold from this set is currently being investigated experimentally.

We evaluated the relative benefit of using an eight-way alignment instead of a pair-wise alignment by redoing the sensitivity experiments and part of the shuffling experiments using only the mouse–human subalignment. The sensitivity on the mixed set of Rfam Seed decreased by 59% and the false-positive rate increased slightly (Table S4). Overall, EvoFold made fewer predictions on the pair-wise alignments.

### New miRNAs among Long Intergenic and Intronic Hairpins

The higher-ranked candidate RNAs in several of the fold classifications are greatly enriched for certain classes of known RNAs. In particular, we see a strong enrichment for known miRNAs among the higher-ranked candidates in the class of long intronic and intergenic hairpins (Tables 2 and 3): 36 of our top 100–ranked long intergenic hairpins and 33 of our top 100 long intronic hairpins are known miRNAs. At the time we first computed our set of 48,479 candidate fRNAs, 157 of them were known miRNAs. Since then 38 more of them have been confirmed to be miRNAs in three recent papers [31–33], giving a total of 195 known miRNAs in this set. Altogether, these three recent papers found 55 new miRNAs from among the 1,181,107 conserved segments that were input to EvoFold; thus, EvoFold's sensitivity was 69% (38/55) on these new miRNAs.

**Table 2:**
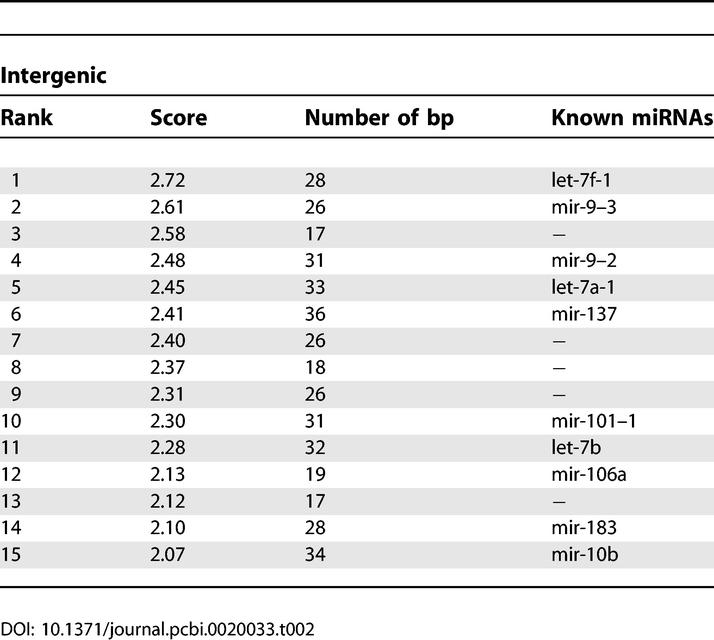
Top-Scoring Long-Intergenic Hairpins

**Table 3:**
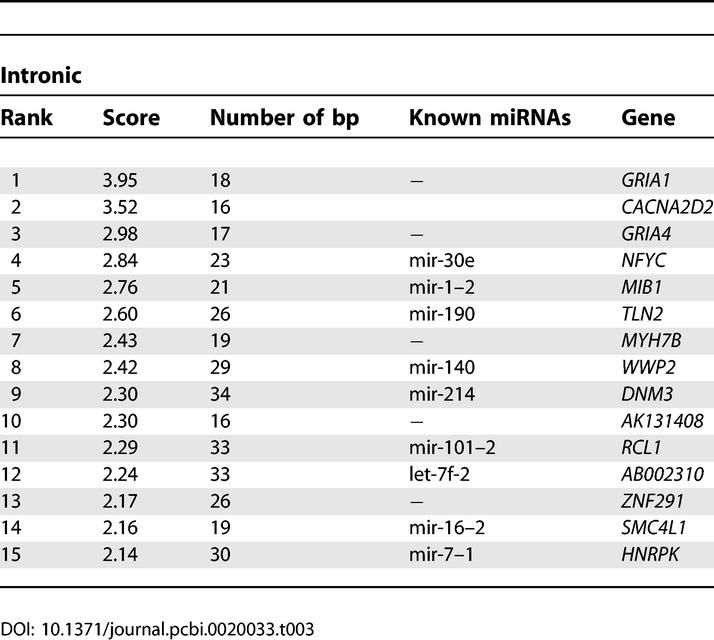
Top-Scoring Long-Intronic Hairpins

The known miRNAs tend to reside in short conserved segments (70% in segments of at most 200 bp), and their stems have relatively few bulges (86% have at most 20% of their bases in bulges). Using these additional criteria we defined a more specific set of 277 miRNA candidates from among the 3,500 predicted long intergenic and intronic hairpins. This set contained 90 known miRNAs and 187 novel candidates, with an estimated false-positive rate of 15% (see Materials and Methods). Xie et al. [31] ended up testing five of our predicted miRNAs and validating four. Bentwich et al. [32] validated 14 of our predicted miRNAs, and Berezikov et al. validated six [33]. Since six candidates were validated multiple times, this gives a total of 18 validated candidates.

While miRNAs probably comprise a significant fraction of the high-scoring intergenic and intronic hairpins, it is quite possible that the majority of the folds in these categories have other functions. In particular, the three highest-scoring long intronic hairpins all are found in introns of ion channel genes, which are frequently targets of RNA editing by A-to-I conversion involving hairpins such as these [34–36]. In A-to-I conversion, the enzyme ADAR (adenosine deaminase acting on RNA), acts on a hairpin RNA structure to change a specific adenosine (A) to inosine (I). One of these genes, *GRIA4,* is already known to harbor an A-to-I editing hairpin in its coding region [37], which we also detected. Thus, there is a possibility that these three intronic hairpins are involved in similar editing on the pre-mRNA.

### New Coding fRNAs

The candidate RNAs contain a surprising number of long folds that overlap coding regions. Coding folds are fascinating for at least two reasons. First, they often function in genetic recoding, which, as in the RNA editing in *GRIA4,* causes the protein made by the ribosome to differ from what would be obtained by a direct translation of the genomic sequence using the genetic code [38]. Second, their primary sequence encodes information both on the protein and the fRNA level, and these dual functional constraints lead to a highly constrained evolutionary process [39].

The 15 top-ranking long-coding hairpins contain eight well-studied RNAs, five of which are involved in genetic recoding in the form of RNA editing (R-G site of *GRIA2, GRIA3,* and *GRIA4*) [37] and programmed frameshifting (*OAZ1* and *OAZ2*) [38,40] (Table 4). Two of the remaining three play roles in regulating translational efficiency *(COL1A1* and *COL1A2)* [41], and one is a miRNA [42,43] overlapping what appears to be a spuriously annotated open reading frame.

**Table 4:**
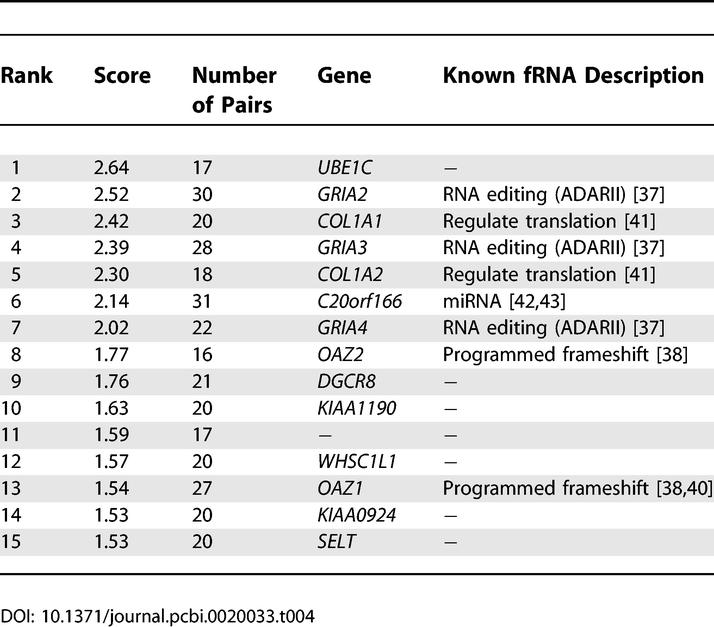
Top-Scoring Long-Coding Hairpins

**Figure 3:**
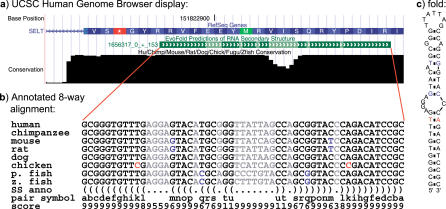
Coding Hairpin near Selenocysteine Insertion Site (A) Gene structure, EvoFold predictions, and conservation around the selenocysteine insertion site of selenoprotein T (SELT). The pairing regions of the hairpin are shown in dark green and can be seen to start only eight bases downstream of the UGA insertion site (indicated by *). Arrows indicate direction of transcription. (B) Annotated segment of eight-way alignment spanning the predicted hairpin. *SS anno,* secondary-structure annotation in parenthesis format (matching parentheses indicate pairs and periods indicate unpaired regions); *pair symbol,* pairing columns are assigned identical symbols to facilitate navigation; *Score,* position-specific scores (0–9), which indicate confidence in secondary-structure annotation. Substitutions in predicted pairs are color-coded relative to the human sequence: green is a compensatory double substitution, blue is a compatible single substitution, and red is a noncompatible substitution. (C) Depiction of hairpin, which is shown with T instead of U to facilitate comparison with the genomic sequences. Pairs are color-coded by presence of substitutions in the eight-way alignment (see b).

Among the seven novel candidate RNAs in the top 15, we predict at least three to be involved in genetic recoding. Two of them are associated with the known selenoproteins *SEPN1* and *SELT* [44]. Selenoproteins constitute another important example of genetic recoding: they contain in-frame UGA stop codons that are recoded as insertion sites for selenocysteines. The recoding of these stop codons is directed by a hairpin called the selenocysteine insertion sequence (SECIS). In eukaryotes the SECIS has previously only been found in the 3′UTR of selenoprotein transcripts [38,44,45], but in prokaryotes it is found in coding regions downstream of the UGA codon [38,46]. Both of these transcripts have an annotated SECIS in their 3′UTR [44,47], but the hairpin structure given in the Rfam database is only partly conserved. The predicted coding hairpins of both *SEPN1* and *SELT* are located less than ten bases downstream from the selenocysteine insertion site (the UGA codon) (Figure 3). We therefore hypothesize that both of these hairpins are involved in the recoding of the UGA codon, and that they may constitute the first examples of Eukaryotic SECIS hairpins in coding regions. During review, we became aware of recent independent experimental work that shows the *SEPN1* hairpin does indeed facilitate UGA readthrough [48].

**Figure 4:**
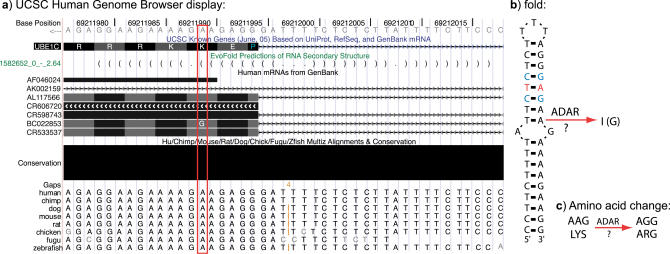
Candidate Substrate for A-to-I Editing (A) Gene structure, EvoFold predictions, cDNAs, conservation, and eight-way alignment are shown at the start of the second exon of the *UBE1C* gene. The predicted hairpin is shown in parenthesis format and can be seen to overlap the intron–exon boundary. The red box highlights a position where the genomic sequence contains an A and a cDNA contains a G. The orange bar and label “4” indicate that up to four extra bases are present in this loop location in the indicated species. (B) Depiction of hairpin (see Figure 3B for color legend) with indication of the potential site of ADAR editing (A-to-I). (C) Which would lead to a lysine to arginine amino acid change.

The third is the highest-ranking long-coding hairpin, found in the *UBE1C* gene (Figure 4). This shows the characteristics of many other hairpins found at sites of A-to-I RNA editing [34–36] by overlapping the intron–exon boundary, and by having a single 1-bp symmetric bulge with consecutive adenosines flanking it. This provides good evidence that this hairpin may function as an A-to-I editing site that is altered in the primary mRNA transcript. An inspection of the human cDNAs spanning this region also revealed a *cDNA* with a single genomic discrepancy showing a guanosine (G) instead of an adenosine (A). Since inosine is sequenced as guanosine, this evidence further supports the hypothesis that this hairpin can function as an A-to-I editing substrate for ADAR.

**Figure 5:**
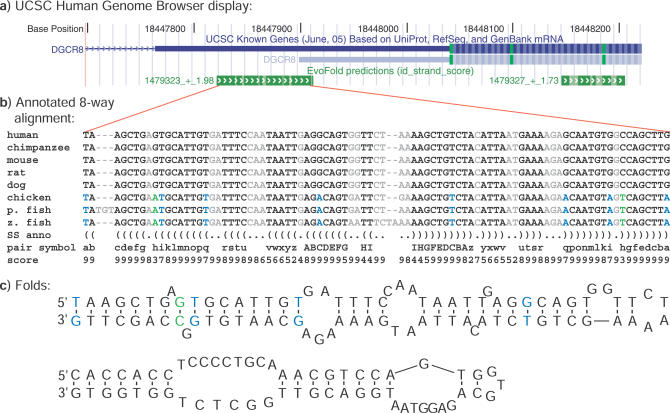
5′UTR miRNA-Like Hairpin and Coding Hairpin in Gene *(DGCR8)* Involved in miRNA Processing (A) Gene structure and EvoFold predictions are shown around the first exon of *DGCR8*. (B) Annotated segment of the eight-way alignment spanning the long, miRNA-like 5′UTR-hairpin (see Figure 3B for legend). (C) Depiction of folds.

Of the four remaining candidate long-coding hairpins, two are in genes of unknown function *(KIAA1190 and KIAA0924),* one is in the Wolf-Hirschhorn syndrome candidate-1 gene, *WHSC1L1* [49], and perhaps the most interesting is in the *DGCR8* (DiGeorge syndrome critical region) gene. The *DGCR8* gene is known to harbor two double-stranded RNA binding domains [50]. *DGCR8* has recently been shown to be associated with Drosha and to play a crucial role in the processing of primary miRNA transcripts to precursor miRNAs [51,52]. This gene harbors not only a high-scoring hairpin in its first exon but also the longest and second highest–scoring hairpin of the 5′UTR category (Figure 5). The 5′UTR hairpin resembles the folds predicted for known miRNAs, and receives a very significant score by mirScan [53] (see Protocol S1). It is therefore possible that these folds are involved in self-regulation of *DGCR8,* potentially through the cleavage of the 5′UTR hairpin by the *DCGR8*/Drosha microprocessor complex described above.

### New Clover-Shaped Folds

**Figure 6:**
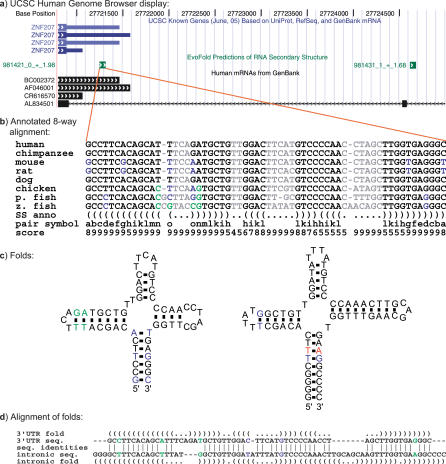
Clover-Shaped Fold Predictions (A) Gene structure, EvoFold predictions, and cDNAs around the end of the gene *ZNF207*. The 3′UTR and the intron of an alternative splice variant harbor high-scoring clover-shaped fold predictions. (B) Annotated segment of eight-way alignment spanning the 3′UTR fold (see Figure 3B for legend). (C) Depictions of 3′UTR fold (left) and intronic fold (right). (D) Annotated alignment of human primary sequences of 3′UTR and intronic folds. The alignment is annotated with the secondary structures of the folds and substitution differences in corresponding pairs are color-coded (see Figure 3B for color legend).

In addition to new examples of previously known RNA families, the high-ranking candidate RNAs also include several completely novel families. One of these is represented by the highest and fourth-highest ranking candidates in the category of long clover-shaped folds. These are located less than 3,500 bases apart, and both are overlapped by transcripts of the little-characterized gene *ZNF207* [54] (Figure 6A). Both folds contain several supporting substitutions (Figure 6B). The shorter of the folds is located in the 3′UTR of the gene and the longer in an intron of an alternative splice variant. The primary sequence of these two folds (Figure 6C) aligns well: the central stem-pairing regions are almost identical with only a few compensatory and compatible substitutions, while the loops differ both by substitutions and insertions/deletions (Figure 6D). This evolutionary relationship suggests a common functional constraint, which has preserved the central part of both clover-shaped folds. The close proximity, the high scores, and the systematic evolutionary differences within as well as between these folds suggest that they may constitute members of a new family of fRNAs.

### Paralogous Families

In the spirit of the last example above, we grouped the RNA-fold predictions into paralogous families based on their primary-sequence homology. We disregarded sequences that could cause homology to be inferred for trivial reasons, i.e., repeats, pseudogenes, coding regions, etc. (see Materials and Methods). This approach resulted in 299 families with a mean family size of 2.7.

Known families of fRNAs were recovered, such as the histone 3′UTR stem loops (46 known folds, one family), families of known miRNAs (72 known folds, 29 families), and families of RNA editing hairpins in GRIA genes (three known folds, one family). But most of the families were completely new. Some contain long intergenic and intronic hairpins and are likely to be new families of miRNAs (e.g., 17 of our miRNA candidates are found in 11 families). Others contain hairpins in ion-channel genes not previously characterized as undergoing RNA editing (e.g., a cluster of three coding hairpins overlapping sodium channel exons in *SCN3A, SCN8A,* and SCN2A2. But the majority involves more complex folds, which we currently have no functional hypotheses for. A complete definition of the families is given online (http://www.cbse.ucsc.edu/jsp/EvoFold).

## Discussion

We have conducted a survey of the human genome to identify functional RNA structures through comparative genomics using an eight-way whole-genome sequence alignment. While this alignment contains considerably more evolutionary information than has been previously available, these currently available genomes are still quite limited in terms of their statistical power to detect negative selection [55], a situation that will change in the coming years as more vertebrate genomes are sequenced. Nevertheless, this study shows that we already have sufficient evolutionary information for efficient discovery of many classes of fRNAs. Further information from additional genomes and additional experiments should be able to weed out many of the false-positive predictions and refine the individual candidate structures.

This initial survey suggests that there are many more functional RNAs in the human genome than are represented in the current RNA sequence databases. We estimate that these databases annotate 1,207 RNA genes in the human genome (see Materials and Methods). Our results suggest that there may be 10-fold more functional RNAs there, and 7-fold more RNA genes. However, these values depend on the ability of the shuffling experiments to correctly estimate the false-positive rate. It is not clear how well shuffling experiments can estimate false-positive rates, and thus our current estimates are associated with very large and difficult to quantify uncertainties. Previous scans for ncRNAs based on pair-wise alignments have found that only a small fraction of the predictions are experimentally verifiable [56,57], thus caution is warranted. Further experimental work will be necessary to reliably characterize the number of human fRNAs. However, combined with the presence of additional evidence (sense-strand bias, transcription evidence, biologically plausible folds, and existence of paralogous families), our results do suggest that there are many additional RNAs to be found. The exploration of RNA genes and RNA structural elements within protein-coding genes represents a huge opportunity, and a huge challenge, as we try to fully explore the key functional elements of the human genome sequence.

The RNA folds we predict with the highest confidence include many known fRNAs, such as miRNAs and genetic recoding signals, as well as thousands of new fRNA candidates, a large fraction of which are supported by the presence of compensatory substitutions. Some of these new fRNAs enlarge existing families while others group into small new families. Detailed analysis of individual candidates has revealed additional supporting evidence and has allowed specific functional hypotheses to be formulated in some cases, including the new SECIS elements, RNA editing hairpins, regulatory hairpins, and miRNA candidates discussed above. We estimate that about 500 coding regions contain overlapping functional RNA structures, and that a non-negligible fraction of these may contain undocumented examples of genetic recoding.

The EvoFold method we have developed was trained to only predict RNA stems that are well-supported by a consistent evolutionary signal in clearly orthologous copies from many species. To guarantee orthology, the alignments used require that aligned sequences from different species appear in the same genomic context, i.e., have orthologous flanking DNA, in each species. This greatly reduces the number of false-positive predictions due to mobile elements such as transposons and retroposed pseudogenes. However, it causes us to miss some highly mobile known fRNAs, such as tRNAs and snoRNAs, even with a relatively liberal threshold that allows an estimated 62% false positives in our overall set of predictions. Identifying mobile fRNAs with a general model of molecular evolution will require logic for lineage-specific duplication and loss of function in addition to the simple evolution of orthologous copies that the EvoFold model embodies.

Alignment errors can also disrupt the evolutionary signal of true fRNAs, and thus improvements to the current sequence-alignment scores might improve the results. Local alignment errors involving only a few bases are unlikely to affect the entire structure and thus should normally allow at least a partial structure with a reduced signal to be identified. However, more extensive errors, where non-orthologous regions are aligned, will most likely cause the fRNA to be missed completely as discussed above.

EvoFold's rate of false positives is much lower among the highest-scoring predictions, but it never goes completely to zero, even for the largest predicted structures. One problem is that the elements where negative selection is strongest, the ultraconserved regions [58], often have too few substitutions within the available vertebrates for the evolutionary approach to distinguish conservation of RNA secondary structure from other kinds of functional conservation. Until more genomes are available, for these elements we are faced with something like the problem of predicting RNA structure in a single sequence, without benefit of comparative genomics.

Sequence comparisons between novel predicted fRNAs verify that some of these can be grouped into small paralogous families, but most appear as singletons. Since many fRNAs undergo lineage-specific expansions [2,32], we find it likely that a search for paralogs in the human genome will show many of these singletons to be founders of phylogenetically shallow families. However, lineage-specific expansion and rapid diversification may make family members difficult to recognize in searches based on primary-sequence identity.

The EvoFold scoring scheme very highly ranks compact folds with a high ratio of paired to unpaired bases, such as miRNAs and histone 3′UTR stem loops. Indeed, these two families stand out prominently in this survey, and their existence would have been a clear-cut new outcome of this study had it not already been known. One of the reasons they rank so highly is because the fps is a length-normalized likelihood ratio, which tends to emphasize the ratio of paired to unpaired bases rather than the total number of paired bases. Other normalization schemes may emphasize other families of fRNAs as shown by the substitution-ranked ncRNA candidates (see Protocol S1).

This set of fold predictions represents what we believe is the first general survey of evolutionarily conserved human fRNAs. (Another survey, based on our multiple alignments and PhastCons detection of conserved segments as well, has come to our attention during the final stages of preparing this paper [59]. The authors appear to have reached similar conclusions regarding the expected number of human RNA genes.) We have attempted to create a comprehensive set, which still maintains a relatively low false-negative rate, in hopes that it would be a useful resource for further studies of fRNAs. To facilitate these further studies, the complete set of predictions is available through the UCSC Human Genome Browser, including detailed structure-labeled alignments as in Figures 3–6 (http://genome.ucsc.edu). Additionally, ranked lists of folds of each category, the set of miRNA candidates, the set of ncRNA candidates, and the set of paralogous families can be accessed from the EvoFold Web site (http://www.cbse.ucsc.edu/jsp/EvoFold).

## Materials and Methods

### EvoFold algorithm.

The EvoFold program takes a multiple alignment and a phylogenetic tree as input, and outputs a specific RNA secondary-structure prediction and an fps (Figure 1). The phylogenetic tree, which includes branch-length estimates, specifies the evolutionary relationship between the sequences of the multiple alignment. EvoFold is based upon two phylo-SCFGs: an fRNA model that describes regions possibly containing fRNAs and a background model that describes regions with no fRNAs. The score is a log-likelihood ratio under these two models. A Linux (i386) executable of the EvoFold program can be downloaded from the EvoFold Web site (http://www.cbse.ucsc.edu/jsp/EvoFold). Source code is available upon request.

### The phylo-SCFGs.

Phylo-SCFGs were developed by Knudsen and Hein in 1999 and can be seen as an extension of phylo-HMMs [60–62]. They combine SCFGs' ability to model RNA secondary structure [18,19,63] with phylogenetic models' [21,22] ability to describe the substitution process along the branches of a tree. One of the strengths of this model construction is that it can handle multiple alignments with any number of sequences and weigh their information content in a way that reflects phylogeny.

Two types of phylogenetic models are used by the phylo-SCFGs: a single-nucleotide model and a di-nucleotide model (Figure 1E). The single-nucleotide model describes the substitution process of the nonpairing regions of the RNA secondary structures (i.e., loops and bulges) as well as the nonstructural regions of the genome. The di-nucleotide model describes the substitution process of the stem-pairing nucleotides. These two models differ in various ways, in particular the single nucleotide model makes many kinds of substitutions relatively likely and the di-nucleotide model strongly favors compensatory substitutions.

The phylo-SCFGs are composed of two components: a structural and a nonstructural one (Figures S5 and S6). The structural component describes structural regions whose first and last bases are paired. Such regions can correspond to a single hairpin or a more complex structure, and will be referred to here as folds (Figure 1D). This component contains both a di-nucleotide and a single-nucleotide phylogenetic model. The nonstructural component describes the regions outside folds and contains only a single-nucleotide phylogenetic model.

The fRNA model contains both the structural and the nonstructural component. In contrast, the background model contains only the nonstructural component. See Protocol S1 for a complete specification of the phylo-SCFG parameterizations.

### Structure and score predictions.

EvoFold uses the fRNA model to assign a specific RNA secondary-structure prediction to an input alignment (Figure 1C). The most probable structure given the information in the multiple alignment will be predicted. A prediction devoid of structure is possible due to the nonstructural component of the fRNA model. All the predicted folds, which pass the fold elimination described below, are included in the candidate set.

The fps measures the overall tendency for the alignment to contain any fRNA. It is calculated as a log-odds score between the likelihood of observing the alignment (*x*) under the fRNA model (*φ_fRNA_*) and the background model (*φ_bg_*): fps = *log*(*P*(*x*|*φ_fRNA_*)/P(*x*|*φ_bg_*). The background model is carefully designed to model alignment sequences using the same nucleotide distribution as the fRNA model, thereby alleviating the problem of overpredicting in, e.g., GC-rich regions. The fps scores are length dependent; length-normalized versions of the fps scores are therefore used in this paper. The scores are used to rank the folds within each subclass.

### Validation.

The false-positive rate of EvoFold was estimated by applying it to a set of alignments that have been randomized to remove the signal of any true fRNAs, but which retain the same base composition, substitution pattern, and conservation pattern as the original alignments. The false-positive rate can be seen to depend on the size of the predicted folds (Figure S2A): ranging from 76% for folds with five or fewer pairing bases to 42% for folds with more than 25 pairing bases. Our set of fold predictions thus contains some false positives, but we decided to retain all but the very short folds to sustain a comprehensive set of folds for downstream analysis. Subsets of folds with a much lower occurrence of false positives can be defined by focusing on only the top-ranked predictions, e.g., there are only an estimated 5% false positives in the top 100 scoring folds with more than 15 bp (Figure S2B and S2C). We also find the false-positive rate to depend on the degree of sequence conservation, the number of bulges found in stems, the genomic location, and to a lesser extent the overall shape of the folds (Figure S3).

### Training data.

The alignments used to train EvoFold were prepared from a conserved subset of the Rfam Full database (version 6.0) [47] as follows: all human entries from Rfam Full were aligned to the human genome using BLAT [64], and only perfect matches were retained. The conserved human–mouse syntenic matching elements (see below) that overlap these human matches were selected and annotated with the secondary structures given in Rfam Full. Annotated stem pairs that could not form in the human sequence were treated as unpaired. Then all tRNA matches were discarded (many were found to be pseudogenes) and alignment sequences with poor secondary-structure conservation were removed. Finally all alignments with fewer than four sequences left were discarded. The resulting set contained 262 annotated alignments. Maximum-likelihood estimates of the phylo-SCFG parameters were found using a combination of the EM algorithm and a quasi-Newton method (see Protocol S1).

### Genomic alignment and conserved elements.

EvoFold was applied to the conserved elements of an eight-way multiz [13] alignment of the following vertebrate species (UCSC assembly designations given in parenthesis): human (hg17), chimpanzee (panTro1), mouse (mm5), rat (rn3), dog (canFam1), chicken (galGal2), fugu (fr1), and zebra fish (danRer1). The PhastCons program [15] was used to identify an initial set of highly conserved elements, which was then processed by joining consecutive elements fewer than 30 bases apart. The joining avoids splitting fRNAs with fast-evolving loop regions across several conserved elements. Since computational constraints limit the size of the elements that can be handled by EvoFold, elements longer than 750 bases were substituted by a tiling of 300 base–long windows each offset by 100 bases. Alignment segments corresponding to both strands of the conserved elements were extracted from the eight-way alignment.

### Phylogenetic tree.

A single phylogenetic tree, including branch lengths, was estimated from the genomic alignment using the PhastCons program [15] and subsequently used with every alignment segment.

### Known fRNA annotations.

The fold predictions were compared against different classes of fRNAs: the 207 human micro RNAs found in the miRNA Registry version 5.1 [43]; the subset of 3′UTR histone stem loops annotated in Rfam Full version 6.0 [47] that overlaps histone-associated transcripts (as defined by the known gene annotation of the UCSC Human Genome Browser [65]); the set of human tRNAs as defined by tRNAscan-SE predictions scoring above 55 bits [66]; the set of snoRNAs defined in snoRNA-LBME-db [67]; and against the more broadly representative set of human fRNAs found in Rfam Seed version 6.0 [47]. When combined, these databases contain a total of 1,207 distinct fRNAs.

### Protein-coding gene annotation.

The known gene annotation from the UCSC Human Genome Browser (May 2004 assembly) [65] was used to annotate the folds with a genomic location. Some folds overlap the boundaries of genomic regions, in these cases a single assignment was chosen according to the following prioritized list: coding > 5′UTR > 3′UTR > intronic > intergenic. The gene names of the known gene track, which are used in Tables 2–4 as well as in the text, are based on RefSeq or HUGO gene symbols.

### Fold elimination.

Folds likely to be nonfunctional based on other annotations, alignments, or genomic location were discarded from the initial set. The filtering comprised certain types of repeats (many trivial folds), regions with synteny breaks (many pseudogenes), and regions homologous to the mitochondrial genome (many pseudogenes). The filters were based on the following UCSC Human Genome Browser data: simple and low-complexity repeats from the RepeatMasker track, synteny information from the mouse net track [68], and homology information from the Blastz self track.

### RNA transcripts.

5′UTR, coding, and 3′UTR folds were considered part of the same transcript if overlapped by a known gene annotation (see above). Intronic and intergenic folds were considered part of the same transcript if separated by fewer than 250 bases. The false-positive rate was estimated from the folds of the relevant genomic types using the randomization procedure described below (see also Validation).

### Randomized alignments.

All input alignments shorter than 450 bases (98% of total) were randomized by first permuting columns with no substitutions and then permuting columns with some substitutions. The resulting alignments thus maintain the conservation pattern, the substitution pattern, and the nucleotide bias of the original alignments, but have lost the signal of any true fRNA stems.

### Paralogous families.

The folds were clustered according to primary-sequence homology, as given by the human Blastz self track of the UCSC browser, thereby defining a set of paralogous families [58]. To avoid inferring homology for trivial reasons, we disregarded sequences annotated as coding, repeats, retro-genes, or pseudo-autosomal regions in the UCSC Human Genome Browser (May 2004 assembly).

## Supporting Information

Figure S1 Length of Folds and Conserved Segments versus Frequency Counts Top, length of folds; bottom, length of conserved segments.
There are 252 folds longer than 250 nucleotides and 1727 conserved segments longer than 1000 nucleotides, which are not included in the above plots.
(18 KB PDF)

Figure S2 Estimated Fraction of False-Positive Predictions (A) Count of false positives for different size-ranges of folds. Black bars indicate number of predictions made in randomized alignments (false positives), gray bars indicate the additional number of predictions made in original alignments (true positives). The estimated fraction of false positives is indicated above each column.
(B and C) Fraction of false positives in different top-score–ranked subsets of short folds (B) and long folds (C). Same color coding as for (A).
(30 KB PDF)

Figure S3 Estimated Fraction of False-Positive Predictions as a Function of Various Fold Properties for Short and Long Folds Left column, short folds; right column, long folds.
For all parts the *x*-axis gives a measure (or type) of the property in question and the *y*-axis gives the corresponding fraction of false positive.
Definition of properties:
(A) The sequence conservation of scores are measured at the input element level and the percentiles are relative to their distribution among all the folds.
(B) The bulge fraction is the percentage of bases in stems found in bulges.
(C and D) The genic location and the fold shape are taken from the fold classification scheme (see Materials and Methods).
(34 KB PDF)

Figure S4 Transcription Evidence for Predicted Folds, Conserved Elements, and Different Classes of ncRNAs The *y*-axis indicates the coverage in percent. The different types of transcription evidence are given along the *x*-axis: TF polyA+, transfags enriched in polyadenylated transcripts; TF polyA−, transfags depleted of polyadenylated transcripts; cDNA, human cDNAs; xeno cDNA, non-human CDNAs; EST, human ESTs; xeno EST, non-human ESTs. The enrichment for a given type of transcription evidence relative to the genome-wide coverage of intronic and intergenic regions is given above each column. The combined class combines the tRNAs, miRNAs, snoRNAs, and the Rfam seed noncoding RNAs.
(28 KB PDF)

Figure S5 Production Rules of the Nonstructural Component and the Structural Component (A) Nonstructural component, (B) structural component.
Nomenclature: | denotes a choice between different productions; x, single column emissions; x_l_ and x_r_, left and right part of pair emissions, respectively.
A corresponding graphical overview of these grammar components are given in Figure S6.
(45 KB PDF)

Figure S6 Transition Graphs of the Nonstructural Component and the Structural Component of the Phylo-SCFGs (A) Nonstructural component, (B) structural component.
The state types are given in parentheses. Arrows indicate possible state transitions. The transition from the bifurcation state leads to two states, a left (l) and a right (r), as indicated on the graph. The unpaired and the loop & bulge states have associated single-column emission distributions (specified by a single-nucleotide phylogenetic model). The stem pair state has an associated di-column emission distribution (specified by a di-nucleotide phylogenetic model).
(31 KB PDF)

Protocol S1 Supplementary Results (176 KB PDF)

Table S1 Count Statistics for Short Fold Classes The fold counts, estimated true positive rate (in parentheses), and estimated true positive counts are given for each location/shape class of short folds. The “any shape” row and the “any location” column give the marginalized counts for each set of fold classes. The entry at the lower right corner thus holds the overall counts for the set of long folds.
(26 KB PDF)

Table S2 Count Statistics for Long Fold Classes See legend for Table S1.
(29 KB PDF)

Table S3 Strand Bias of EvoFold Predictions (33 KB PDF)

Table S4 EvoFold Sensitivity Using Only Human and Mouse Sequences The sensitivity column gives the number of known fRNAs recognized by EvoFold using the human–mouse subalignments divided by the total number of fRNAs in the input segments. The relative sensitivity column gives the ratio between the sensitivity using only the human and mouse subalignment and the complete eight-way alignment.
(147 KB PDF)

### Accession Numbers

Accession numbers from Swiss-Prot (http://www.ebi.ac.uk/swissprot) are: *COL1A1* (P02452), *COL1A2* (P08123), *DGCR8* (Q8WYQ5), *GRIA2* (P42262), *GRIA3* (P42263), *GRIA4* (P48058), *KIAA1190* (Q6ZSY6), *KIAA0924* (Q5H9Q0), *OAZ1* (P54368), *OAZ2* (O95190), *SCN2A2* (Q99250), *SCN3A* (Q9NY46), *SCN8A* (Q9UQD0), *SEPN1* (Q9NZV5), *SELT* (P62341), *UBE1C* (Q8TBC4), *WHSC1L1* (Q6ZSA5), and *ZNF207* (O43670).

The GenBank (http://www.ncbi.nlm.nih.gov/Genbank) accession number for *cDNA* of UBE1C gene is BC022853.

## Abbreviations

ADAR: (adenosine deaminase acting on RNA
bp: base pair
*DGCR8*: DiGeorge syndrome critical region
fps: folding potential score
fRNA: functional RNA structures
phylo-SCFG: phylogenetic stochastic context-free grammar
SECIS: selenocysteine insertion sequence
UCSC: University of California Santa Cruz
