# Supplementary material for: Identification and Classification of Conserved RNA Secondary Structures in the Human Genome
Source: PLoS Comput Biol. 2006 Apr 21;2(4):e33. doi: 10.1371/journal.pcbi.0020033 (PMC1440920; doi:10.1371/journal.pcbi.0020033)
Supplement: Supplementary file 1 [file pcbi.0020033.sd001.pdf]

Identification and Classification of Conserved RNA Secondary Structures in the Human Genome

Supplementary material

Jakob Skou Pedersen[1*], Gill Bejerano[1], Adam Siepel[1], Kate Rosenbloom[1], Kerstin Lindblad-Toh[3], Eric S. Lander[3], Jim Kent[1], Webb Miller[4] and David Haussler[1,2]

[1] Center for Biomolecular Science and Engineering, [2] Howard Hughes Medical Institute, University of California, Santa Cruz, Santa Cruz, California 95064, USA. [3] Broad Institute of MIT and Harvard, Cambridge, Massachusetts 02141, USA. [4] Center for Comparative Genomics and Bioinformatics, Pennsylvania State University, University Park, Pennsylvania 16802, USA.

[*] To whom correspondence should be addressed. e-mail: jsp@soe.ucsc.edu

Supplementary results

Sense-strand bias of EvoFold scores

Further assessment of the efficacy of the EvoFold algorithm can be made by noting that its score is not strand-symmetric. The asymmetry is primarily caused by the ability of GU (or UG) to pair, but not its reverse complement AC (CA). Since the most common types of substitution in RNA stems involve GU (or UG) pairs, this effect can have a pronounced effect on the EvoFold score, thus allowing the strand association of a fold to be inferred by comparing the score of an alignment with the score of its reverse complement. Since genes are expected to harbor fRNAs in their UTRs and to a lesser extent in their introns, the true folds should show a tendency to correlate with the transcribed strand. A highly significant correlation is indeed observed in these regions, ranking as one would expect: 3'UTR > 5' UTR > intronic. Curiously, the coding regions show a tendency to be associated with the reverse strand (Table S1).

EvoFold was used to make predictions on both the sense (i.e. the strand complementary to the template strand for transcription) and the anti-sense strands of protein-coding gene transcripts. The folding-potential score of each predicted fold was subsequently evaluated on both strands and assigned a strand-

Table S1: Strand bias of EvoFold predictions

Genic region	count	ass. statistic	P-value [b]
coding	10551	0.496	0.47
5'UTR	207	0.553	0.164
3'UTR	2725	0.646	$< 2.2e-16$
intron	9603	0.558	$< 2.2e-16$
combined	23086	0.549	$< 2.2e-16$

[b] The association statistic was assumed to be binomial distributed with parameter p=0.5. The alternative hypothesis is that p deviates from 0.5.

preference score: one if the sense strand scored highest, 0.5 if both strands scored equally, and zero if the anti-sense strand scored highest. These statistics were then used to calculate the degree and significance of association with the sense strand (Table S1). Only folds completely embedded within a given genic region were used. The Known Genes track of the UCSC Browser was used to define the genic regions (see main text, methods).

The conserved elements of all the genic regions, apart from the coding, have a compositional bias toward G and T (coding: 48.2%, 5'UTR: 51.2%, 3'UTR: 50.7%, and intron: 52.1%, based on 1000 random samples of each type). Such a compositional bias has previously been obsersved on the sense-strand of transcribed regions and has been hypothesized to be caused by a repair mediated substitution bias ([1]). However, the conserved elements we analyze are under negative selection, which appears to be a stronger force in shaping their composition, as coding regions are slightly depleted for G and T and the stems of our train set further enriched (55.1% G and T). In comparison, the overall G and T content of the complete candidate set is 53.0%. The observed biases toward G and T could be caused by encoded RNA structures, but could also be caused by the encoding of some other type of G and T rich functional elements. A high G and T content makes substitutions more likely to be consistent with the prediction of RNA structure. Hence, even though the observed correlation is consistent with a surplus of functional RNAs on the sense strand of transcribed regions, we cannot rule out that the observed sense-strand correlation is the effect of an unrelated functional bias toward high G and T content nor that it is influenced by the compositional bias observed by Green et al..

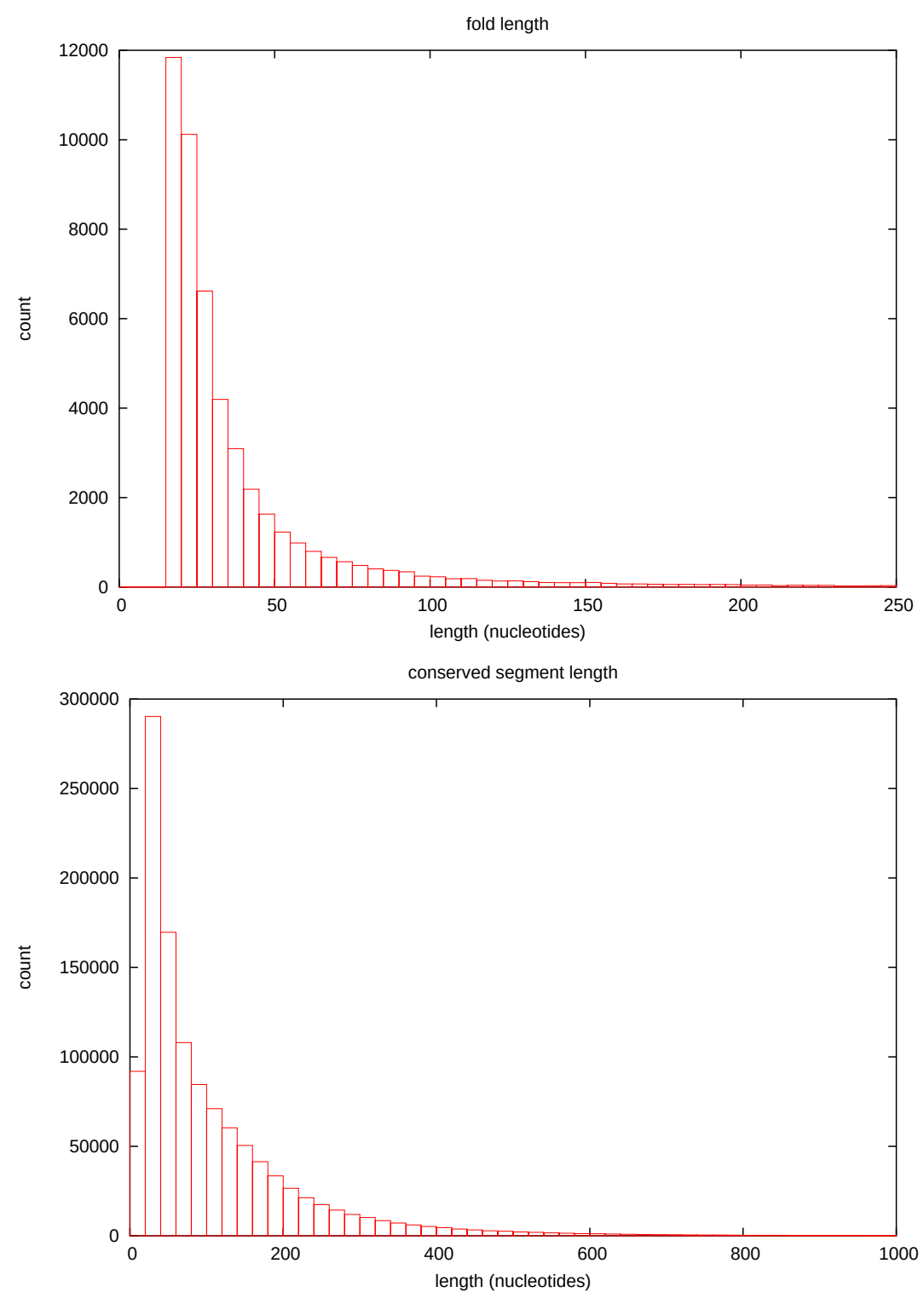

Figure S1: Length of folds (top) and conserved segments (bottom) versus frequency counts. There are 252 folds longer than 250 nucleotides and 1727 conserved segments longer than 1000 nucleotides, which are not included in the above plots.

Table S2: Count statistics for short fold classes

type\location	5'UTR			3'UTR			coding			intronic			intergenic			any location		
hairpin	263	(20%)	53	2567	(44%)	1139	10945	(20%)	2217	8965	(41%)	3668	15667	(42%)	6538	38407	(35%)	13614
Y-shaped	3	(67%)	2	49	(11%)	5	327	(30%)	99	120	(50%)	60	160	(33%)	52	659	(33%)	219
clover-shaped	0	(n.a.)	0	0	(n.a.)	0	6	(80%)	5	1	(0%)	0	1	(100%)	1	8	(75%)	6
complex shapes	0	(n.a.)	0	0	(n.a.)	0	0	(n.a.)	0	1	(0%)	0	0	(n.a.)	0	1	(0%)	0
any shape	266	(21%)	55	2616	(44%)	1144	11278	(21%)	2321	9087	(41%)	3728	15828	(42%)	6591	39075	(35%)	13839

The fold counts, estimated true positive rate (in parenthesis), and estimated true positive counts are given for each location/shape-class of short folds. The "any shape" row and the "any location" column gives the marginalized counts for each set of fold classes. The entry at the lower right corner thus holds the overall counts for the set of long folds.

Table S3: Count statistics for long fold classes

type\location	5'UTR			3'UTR			coding			intronic			intergenic			any location		
hairpin	32	(56%)	18	359	(37%)	132	666	(36%)	237	1262	(56%)	702	2238	(59%)	1325	4557	(53%)	2413
Y-shaped	20	(0%)	0	205	(36%)	74	548	(39%)	213	803	(49%)	397	1244	(50%)	623	2820	(46%)	1307
clover-shaped	4	(0%)	0	17	(100%)	17	39	(37%)	14	71	(61%)	43	111	(69%)	77	242	(63%)	152
complex shapes	12	(33%)	4	134	(29%)	39	205	(39%)	81	554	(55%)	307	880	(46%)	402	1785	(47%)	833
any shape	68	(32%)	22	715	(37%)	261	1458	(37%)	546	2690	(54%)	1449	4473	(54%)	2427	9404	(50%)	4705

See legend of Table S2.

Estimating false positive rates for EvoFold

The false positive rate of prediction was assessed through shuffling (randomization) experiments as explained in the fold analysis subsection of the materials and methods section of the main text. The shuffling was done for each conserved element separately and the predicted folds were annotated with their size, genomic location, and fold shape, thereby allowing the false positive rate (and hence the true positive rate) to be estimated for each fold class. A subset of the conserved elements span several genomic regions. Since the shuffling procedure preserves the conservation pattern along the alignments, the predictions in shuffled alignments are assigned genomic locations based on the locations of genomic regions in the original alignments. Table S3 and Table S2 give the raw fold count, the estimated true positive rate, and the estimated true positive count within each class of long and short folds, respectively.

The accuracy of the true positive estimates are dependent on the abillity of the shuffling approach to generate alignments with the same folding potentials as the originals. Although care has been taken to achieve this, significant differences may still persist. The estimates of true positive rates (and thereby true positive counts) are therefore affiliated with huge uncertainties and should be interpreted with caution. Furthermore, studies based on pairwise genomic scans have found only a small fraction of the predictions to be verifiable [2, 3]. Some of the defined fold classes contain very few folds (and some none), which will add a further level of uncertainty to their false positive rate estimates. Albeit the above stated reservations, we still believe the true positive estimates are indicative of the fraction of true positives and that they can be meaningfully compared between classes.

The estimated false positive rate for the complete set is around 62%. However, the estimates varies greatly between the different classes as well as with score-rank, level of conservation, and the fraction of bulges in the stems of the folds (see fig.S2 and fig.S3.

The short folds (<15 pairing bases) have the highest false positive rate and make up the majority (81%) of the folds, and therefore dominate the overall estimates (see fig. S2a). The estimated false positive rates of the highest ranking folds are much lower than the average (see fig. S2b,c). This coincides well with the high proportion of annotated and biologically plausible folds found among the top-15 ranking folds of various classes of long hairpins (see Table 2 and 3 of main text).

The false positive rate is strongly dependent on the level of conservation of the input elements and attains levels around 80% for both short and long folds in extremely conserved regions (see fig. S3a). The absence of substitutions in the highly conserved regions will often allow spurious stems to be predicted without a contradictory evolutionary signal. On the other hand, In the least conserved half of the input elements, the estimated false positive level is significantly below average (around 34%), due to fewer a stronger evolutionary signal.

The false positive rate also varies with the fraction of bulges in stems, albeit not as significantly as for, e.g., conservation (see fig. S3b). For the long folds,

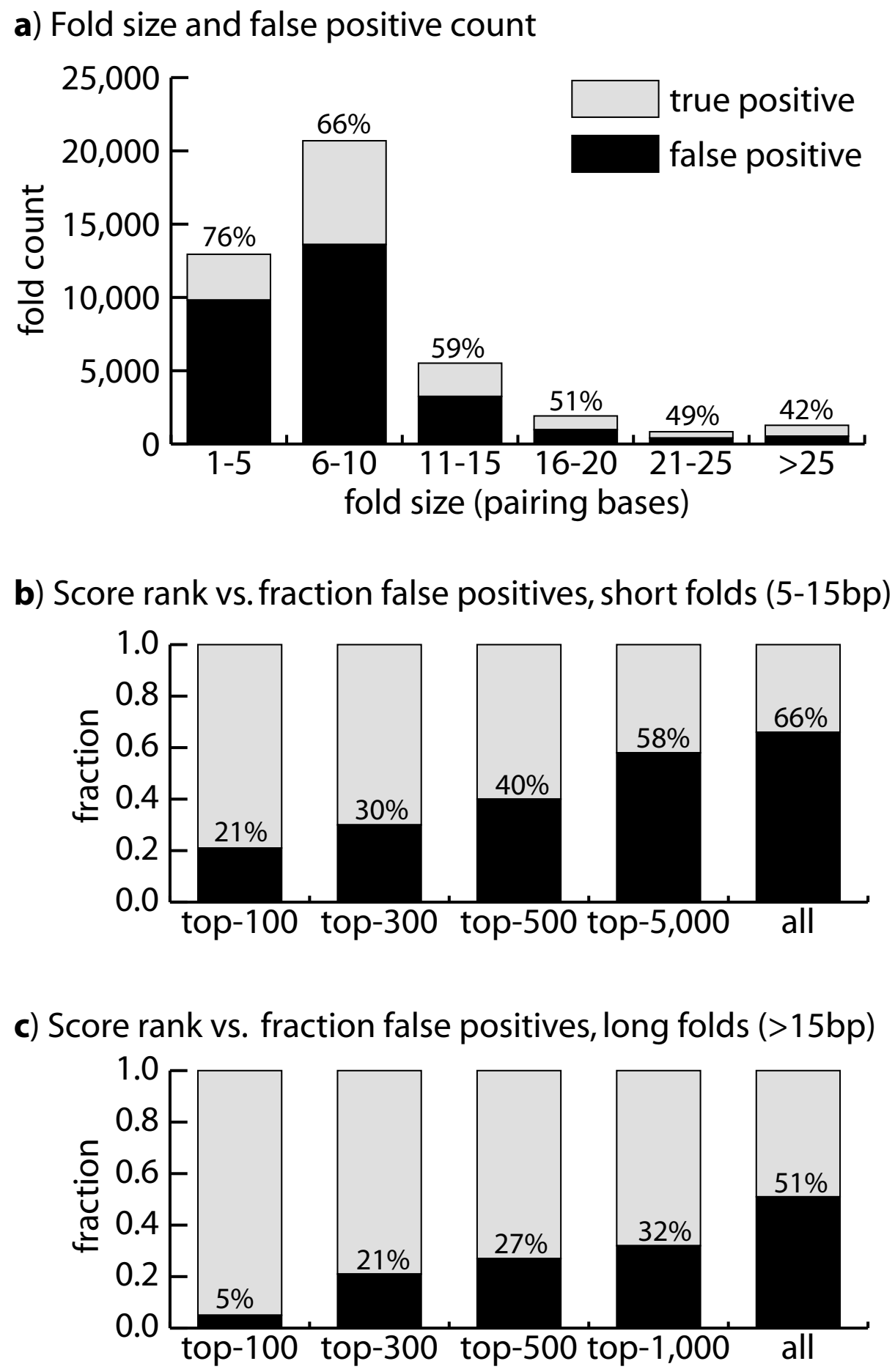

Figure S2: Estimated fraction of false positive predictions. **a**: Count of false positives for different size-ranges of folds. Black bars indicate number of predictions made in randomized alignments (false positives), gray bars indicate the additional number of predictions made in original alignments (true positives). The estimated fraction of false positives is indicated above each column. **b,c**: Fraction of false positives in different top-score-ranked subsets of short folds (fig. b) and long folds (fig. c). Same color coding as for fig. a.

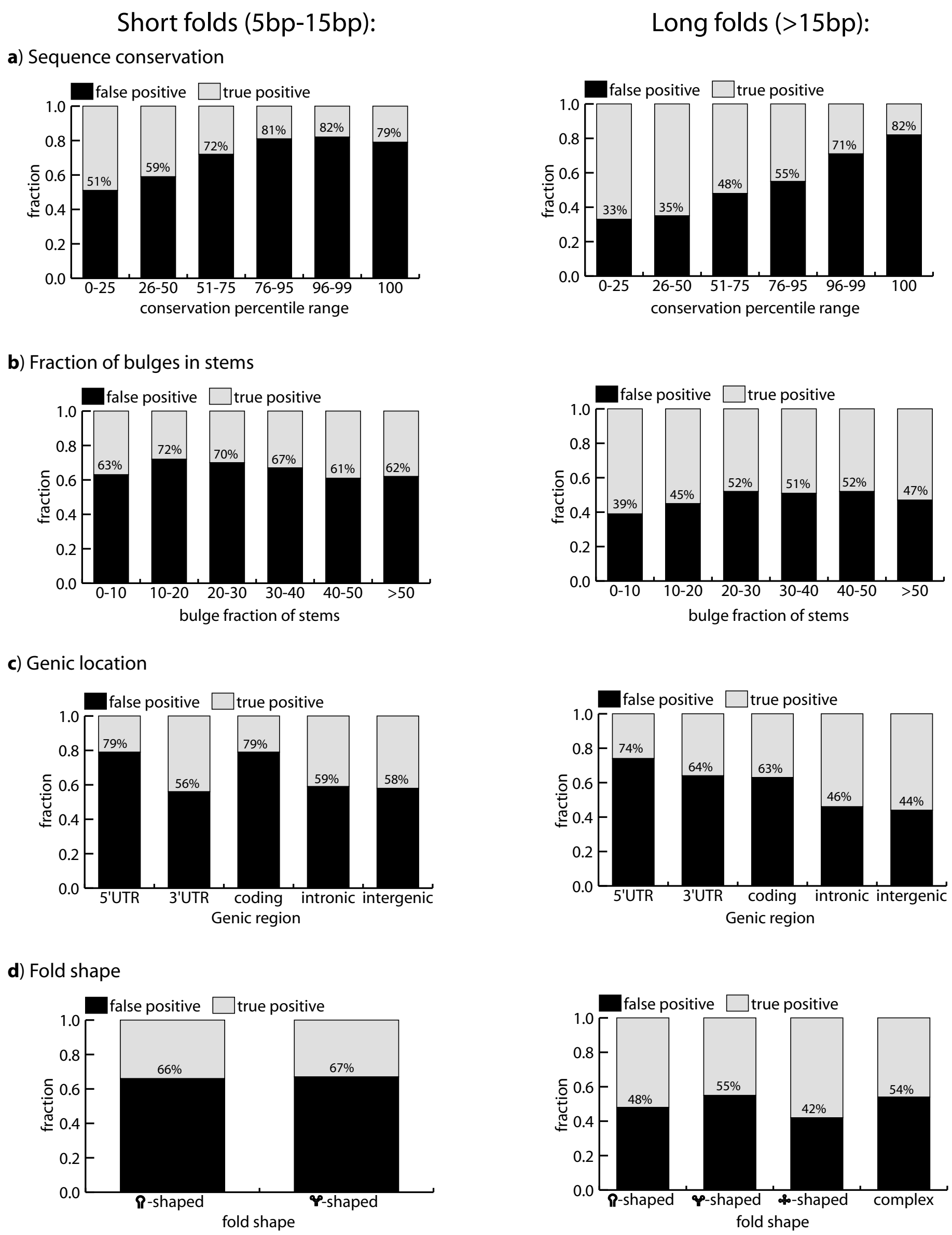

Figure S3: Estimated fraction of false positive predictions as a function of various fold properties for short (left) as well as long (right) folds. For all figures the x-axis gives a measure (or type) of the property in question and the y-axis gives the corresponding fraction of false positive. Definition of properties: **a**: The sequence conservation scores are measured at the input element level and the percentiles are relative to their distribution among all the folds. **b**: The bulge fraction is the percentage of bases in stems found in bulges. **c and d**: The genic location and the fold shape is taken from the fold classification scheme (see methods section of main text for definitions).

structures with few bulges achieve the lowest false positive rate.

Genic location also affects the false positive rate, with the highest estimates found for coding and 5'UTR regions (see fig. S3c). Only hairpins and Y-shaped folds are present at noticable amount among the short folds, and they achieve uniformly high false positive rates (around 66%) (see fig. S3d). Interestingly, the clover-shaped folds achive the lowest false positive rate (42%) among the long folds.

Transcription evidence for intergenic folds

We measured the genomic coverage (nucleotide level) of our intergenic and intronic folds by different types of transcription evidence and compared it to the coverage of conserved elements in the same genic regions (fig. S2d). The folds show an enrichment for human as well as non-human cDNAs and ESTs relative to the conserved elements, and both folds and conserved elements are enriched relative to the background coverage in these genic regions. The enrichment for the folds relative to the background ranges from 3.6x (human cDNA) to 11.4x (non-human EST) while the enrichment of the conserved elements relative to background ranges from 2.7x (human cDNA) to 7.3x (non-human EST).

For comparison we also analyzed the coverage of transcription evidence in different classes of known fRNAs in intronic and intergenic regions (fig. S4). The observed enrichments differ widely between the different classes of fRNAs: snoRNAs achieve the highest enrichments ranging from 8.6x (human ESTs) to 32.4x (non-human EST) and tRNAs the lowest enrichments ranging from 0.0x (non-human cDNA) to 4.1x (human EST). The transcription evidence coverage depends on many factors, such as transcript abundance, transcript purification procedures, masking of repetitive elements, ascertainment for known fRNAs, copy number of fRNAs, cross hybridization, etc., and it is therefore not possible to estimate the fraction of true fRNAs in the fold predictions from their coverage by transcriptional evidence.

Substitution-ranked ncRNA candidates

The folding potential score highly ranks some types of fRNAs, particularly ones with deeply conserved compact folds, but not some other types. In order to define a set enriched for some of these other types of fRNAs, we defined a score directly based on the observed substitutions and used it to rank long (¿15 pairs) intronic and intergenic folds. Only folds with less than 50% bulges in their stems were considered.

The score is a normalized linear combination of the number of compensatory double substitutions (x), compatible single substitutions (y), and contradictory substitutions(z) observed in the input alignment:

$$\text{score} = (x + 0.25y - 0.5z)/\text{no. pairs}$$

This score favors folds with many compensatory substitutions and few contradictory substitutions.

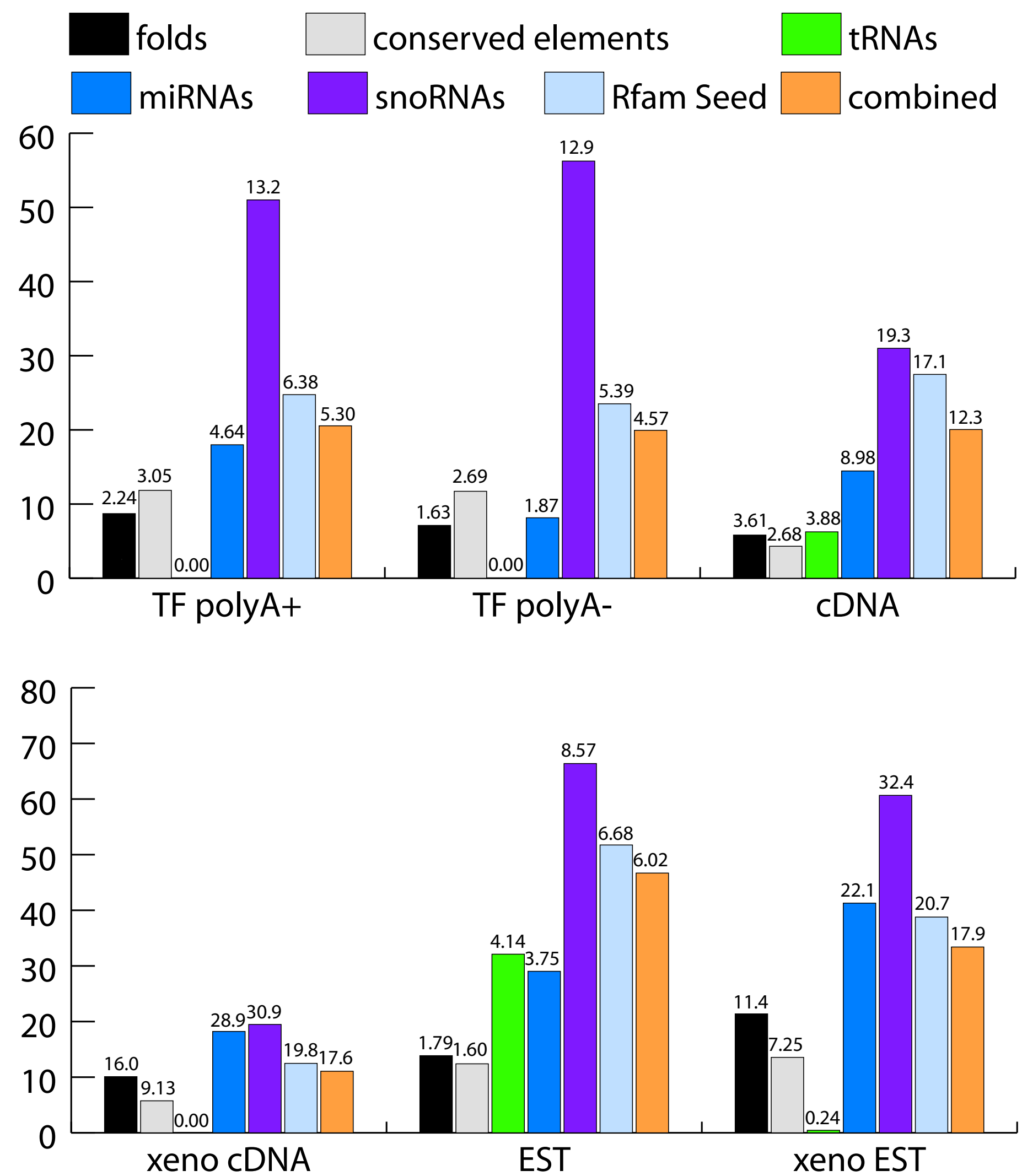

Figure S4: Transcription evidence for predicted folds, conserved elements, and different classes of ncRNAs. The y-axis indicates the coverage in percent. The different types of transcription evidence are given along the x-axis: **TF ployA+**: Transfrags enriched in polyadenylated transcripts, **TF polyA-**: Transfrags depleted of polyadenylated transcripts, **cDNA**: human cDNAs, **xeno cDNA** non-human cDNAs, **EST**: human ESTs, **xeno EST**: non-human ESTs. The enrichment for a given type of transcription evidence relative to the genome-wide coverage of intronic and intergenic regions is given above each column. The combined class combines the tRNAs, miRNAs, snoRNAs, and the Rfam Seed non-coding RNAs.

Table S4: EvoFold sensitivity using only human and mouse sequences

Data set	sensitivity		relative sensitivity	
miRNA registry [4]	48%	(88/183)	56%	(88/157)
Histone 3'UTR stem-loops [5]	0%	(0/64)	0%	(0/62)
snoRNAs [6]	2%	(4/190)	40%	(4/10)
tRNAs [7]	0%	(0/2)	0%	(0/2)
Rfam Seed [5]	18%	(41/231)	41%	(41/100)

The sensitivity column gives the number of known fRNAs recognized by EvoFold using the human-mouse sub-alignment divided by the total number of fRNAs in the input segments. The relative sensitivity column gives the ratio between the sensitivity using only the human and mouse sub-alignment and the complete 8-way alignment.

A set of 517 top-ranking folds (hairpins, Y-shaped, and clover-shaped) were defined. The thus defined set of ncRNA candidates represent an alternative to the ranked sets we present from the overall classification scheme. The set is, as is the case for all candidate sets, available from the EvoFold web-site (`http://www.cbse.ucsc.edu/~jsp/EvoFold`).

Performance on pairwise alignment

The benefit of using a multiple alignment instead of just a pairwise alignment was assessed by redoing sensitivity and specificity experiments on pairwise human-mouse alignments extracted from the multiple alignments. The original input data was used such that these experiments differ only by the number of sequences in the alignments.

Pairwise sensitivity

The five-fold cross evaluation described in the main text was repeated on the pairwise alignments. The training was done on the 8-way alignments, while the testing was done on the pairwise alignments.

The decrease in performance varies between different types of functional RNAs (see Table S4). The miRNAs maintain little more than half of the sensitivity obtained with the full 8-way alignment, snoRNAs little less than half, while no tRNAs or histone 3'UTR stem-loops are detected. The sensitivity on the mixed set of Rfam Seed fRNAs falls to 41% of the level obtained on the full alignments.

It is interesting to note that the histone 3'UTR stem-loops are identified very efficiently using the 8-way alignments but missed entirely using the pairwise human-mouse alignments. This can be attributed to their short stems, which require a strong evolutionary signal to be detected.

Pairwise specificity

Because of the high computational costs, the specificity experiments were only performed on Chromosome 22, which spans 1.7% of the genome. The overall false positive rate estimated on the mouse-human alignments was 58% (63/109), which is slightly higher than the 54% (300/552) estimated from Chromosome 22 using the 8-way alignment. The false positive estimates for the long folds are based on sparse data but exhibit the same pattern: 30% (3/10) using the the mouse-human alignments and 26% (19/74) using the 8-way alignments.

Both the sensitivity and the specificity experiments reveal that EvoFold makes fewer predictions when little evolutionary information is available. Probably because, with few sequences the cost of predicting a structure by the SCFG will often not be compensated by the evolutionary signal (supporting substitutions or conservation).

MirScan evaluation of DGCR8 5'UTR hairpin

The DGCR8 transcript contains the second-highest-scoring long 5'UTR hairpin. This hairpin strongly resembles our predictions for known miRNAs and we therefore evaluated its miRNA potential using MirScan [8].

A 100-bases-long human region (chr22:18447817-18447917) including the fold was extracted from the 8-way alignment together with the homologous sequence from fugu:

```
human TCACTTAAGCTGAGTGCATTGTGATTTCCAATAATTGAGGCAGTGGTTCT
fugu  TCCCGTAAGCTGAATGCATTGTGATTTCCAATAATTGAGACAGTGATTCT

      AAAAGCTGTCTACATTAATGAAAAGAGCAATGTGGCCAGCTTGACTAAGC
      AAAAGCTGTCTACATTAATGAAAAGAACAATGTAGTCAGCTTAGCGTTTT
```

This alignment was given as input to the MirScan Web Server (`http://genes.mit.edu/mirscan/`), which assigned it a score of 14.24. This score is highly significant according to the histogram presented in fig. 1 of Lim et al. (2003) [8].

EvoFold specification

EvoFold is based on a pair of phylo-SCFGs (phylogenetic stochastic context-free grammars) as outlined in the "Materials and methods" section of the main text. This section will start with a brief introduction to SCFGs and then give more detail on the phylogenetic models and the grammars which make up the phylo-SCFGs. A brief description of the algorithms used with the phylo-SCFGs is also included.

SCFGs

SCFGs are probabilistic models which define distributions over sequences with associated structure annotations. SCFGs can be seen to extend HMMs (hidden Markov models): not only can they model neighbor dependencies but also far-ranging nested dependencies. This last property makes them well-suited for modeling the nested, far-ranging base-pair interactions of RNA secondary structures.

SCFGs originate from formal grammars [9, 10] and have traditionally been described as generative models in the terminology of production rules operating on non-terminals and terminals. The non-terminals represent the underlying structural annotation of a sequence. The terminals, on the other hand, represent the observed symbols of a sequence. This formalism does not normally separate the modeling of the underlying structure (involving only non-terminals) from the modeling of the observed sequence (involving both non-terminal and terminals). But in the case of phylo-SCFGs such a separation becomes convenient and allows for a more compact representation of the model [11].

By adopting the terminology of states, transitions, and emission distributions, traditionally used with HMMs, a conceptual separation of the modeling of non-terminals from terminals becomes possible. A state corresponds to a non-terminal, a transition defines the probability of generating a given non-terminal from the current non-terminal, and an emission-distribution defines a probability distribution over all the possible productions of terminals given a certain non-terminal. This approach is described in Durbin et al. as well as Pedersen et al. (2004) and will only be outlined below.

Durbin et al. (1998) suggested the use of a limited number of state types for RNA-grammars, each of which can only be used with a certain form of production rule: pair (P) ($W_v \rightarrow x_i W_y x_j$), left (L) ($W_v \rightarrow x_i W_y$), start (S) ($W_v \rightarrow W_y$), bifurcate (B) ($W_v \rightarrow W_y W_Z$), and end (E) ($W_v \rightarrow \epsilon$), where ϵ denotes the empty sequence, W_v, W_y and W_z denote different states, x denotes the observed sequence, and i and j positions within the observed sequence .

Given this set of possible production rules an SCFG can be completely specified by a four-tuple $\phi = (W, t, A, e)$, where W is a set of states, t is a set of state transitions, A is a set of state-associated emission distributions, and e is a set of state-associated emission distributions [11]. Alphabets and emission distributions are only defined for the subset of emitting states, which are either of type *pair* or type *left*.

In the case of phylo-SCFGs, the alphabets are composed of alignment columns and the emission distributions are defined by phylogenetic models.

Phylo-SCFGs

EvoFold is based on two phylo-SCFGs: an fRNA model (ϕ_{fRNA}), which models regions with functional RNA structures, and a background model (ϕ_{bg}), which models region without any structures. The fRNA model is composed both of a non-structural component (fig. S5a and fig. S6a), a structural component

a)

$$\begin{array}{rcl} \text{begin\_non-structrual} & \rightarrow & \text{unpaired} \\ \text{unpaired} & \rightarrow & \text{unpaired } x \mid \text{end } x \end{array}$$

b)

$$\begin{array}{rcl} \text{begin\_structrual} & \rightarrow & \text{stem\_pair} \\ \text{stem\_pair} & \rightarrow & x_l \text{ stem\_pair } x_r \mid x_l \text{ bifurcation } x_r \\ \text{bifurcation} & \rightarrow & \text{emit intermediate} \\ \text{emit} & \rightarrow & \text{loop\_\&\_bulge } \mid \text{stem\_pair} \\ \text{loop\_\&\_bulge} & \rightarrow & \text{end } x \\ \text{intermediate} & \rightarrow & \text{bifurcation} \mid \text{emit} \end{array}$$

Figure S5: Production rules of the non-structural component (**a**) and the structural component (**b**). Nomenclature: '|' denotes a choice between different productions; x denotes single column emissions; x_l and x_r denotes the left and right part of pair emissions, respectively. A corresponding graphical overview of these grammar components are given in fig. S6.

(fig. S5b and fig. S6b), and a high-level component (not shown), which combines the two other components. In contrast, the background model consists of only the non-structural component.

The overall structure of the fRNA grammar is identical to the grammar used by RNA-decoder [11], but the structural and the non-structural components are different. The structural component uses the RNA-grammar proposed by [13]. Note that we write these grammars using the restricted set of production rules given above, this causes the structural grammar to be written with three more states than originally used by Knudsen and Hein. A minimum loop-size of three bases is enforced by the emission distribution of the *stem pair* state. No minimum stem size is enforced.

Phylogenetic models

Since the input of EvoFold is multiple sequence alignments, the emission distributions of its SCFGs are defined over sets of alignments columns. There are exceedingly many such alignment columns for even a moderate number of aligned sequences: 16^n in the case of paired columns with n sequences in the alignment. Explicitly defining a distribution over these columns is therefore infeasible.

The use of phylogenetic models for studying molecular evolution and evolutionary relationships has a long history [14, 15], and they are now becoming

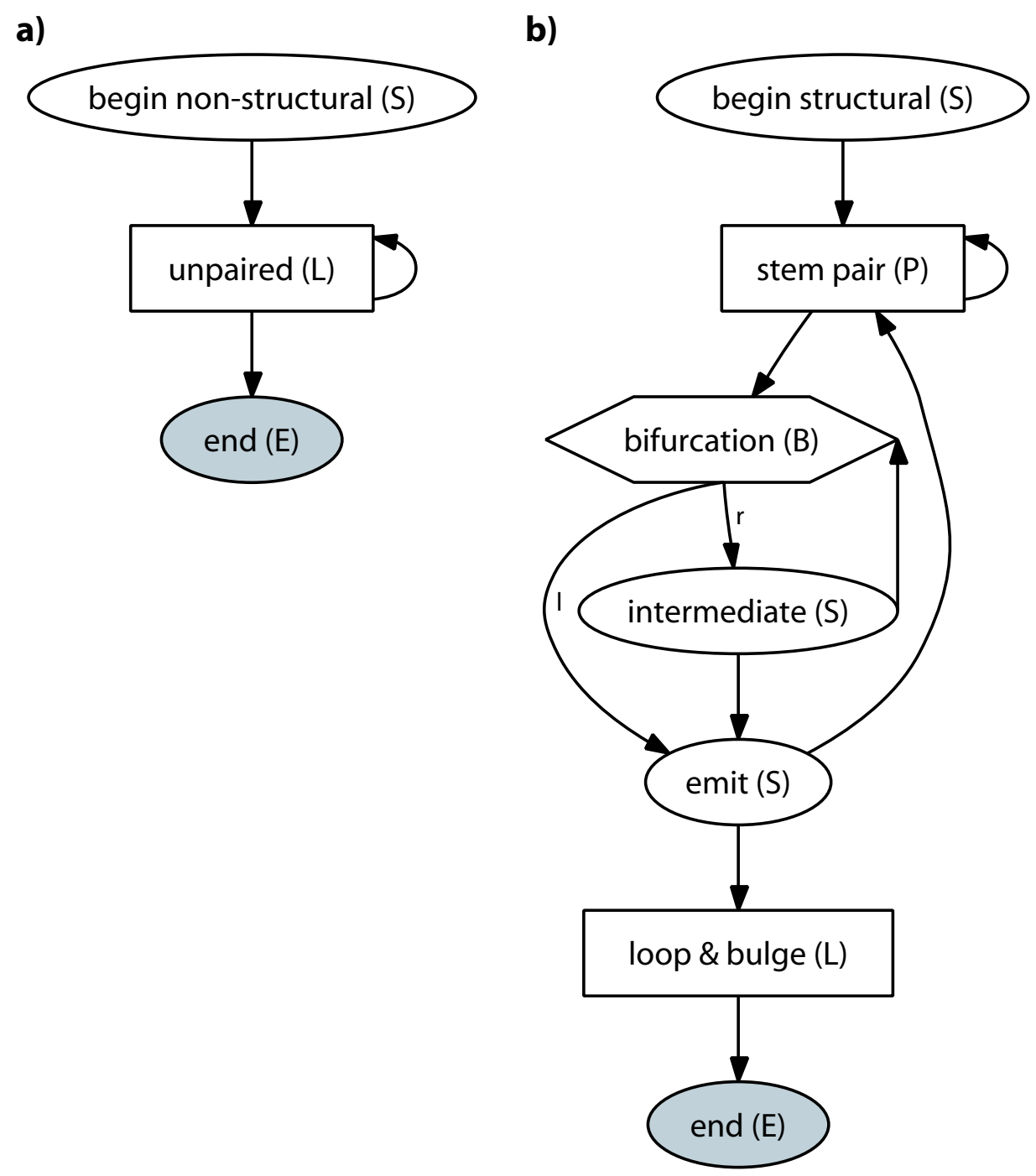

Figure S6: Transition graphs of the non-structural component (**a**) and the structural component (**b**) of the phylo-SCFGs. The state types are given in parenthesis. Arrows indicate possible state transitions. The transition from the *bifurcation* state leads to two states, a left (l) and a right (r), as indicated on the graph. The *unpaired* and the *loop & bulge* states have associated single-column emission distributions (specified by a single-nucleotide phylogenetic model). The *stem pair* state has an associated di-column emission distribution (specified by a di-nucleotide phylogenetic model).

an integral model component in comparative genomics [13, 16–18]. They describe the substitution process along the branches of a tree, and can be used to efficiently calculate the probability of any given alignment column. They are normally highly parameterized but still able to capture characteristics of the substitution process, such as rate of substitution, nucleotide bias, and unequal rates of substitution.

A phylogenetic model is defined by $\psi = (Q, \tau, \beta)$, where Q is an instantaneous rate matrix, τ is a tree topology, and β is a set of branch lengths. Phylogenetic models are normally based on a stationary, reversible continuous-time Markov process. Stationarity implies that the initial distribution of the process equals the equilibrium distribution π. Reversibility is normally achieved by parameterizing Q in terms of pi. The resulting process is defined with respect to a set of states, which in our case is represented by a nucleotide or a di-nucleotide alphabet. The tree topology and the set of branch lengths specify the phylogenetic tree.

The fRNA model makes use of two emission distributions: The two left emitting states (*unpaired* and *loop & bulge*) use the same emission distribution, which is specified by a single-nucleotide phylogenetic model (ψ^{single}). The pair-emitting state (*stem pair*) uses an emission distribution defined by a di-nucleotide phylogenetic model (ψ^{di}). Since the background model is defined as the non-structural component of the fRNA model, it uses the same single-nucleotide model.

Phylogenetic tree

The phylogenetic tree represents the evolutionary relationship between the sequences (i.e. species) of the multiple alignment. It is specified by both a topology and a set of branch lengths, as stated above. The branch lengths are measured in expected number of nucleotide substitutions per site. By increasing the branch lengths, alignment columns with many substitutions become more likely. The phylogenetic trees of the two phylogenetic models are based on the same input tree, but their branch lengths are scaled differently (e.g., $\beta = r^{pair}\beta^{input}$, where r^{di} is the scaling factor used for the di-nucleotide model). The branch lengths of the di-nucleotide are down-scaled with a factor of 2.4 relative to the single-nucleotide model (i.e., $r^{single} = 2.4r^{di}$).

The phylogenetic tree is estimated using the phastCons model of the PHAST software package `http://www.soe.ucsc.edu/~acs/software.html` [19]. The phastCons model was estimated from the genome-wide 8-way alignment (see materials and methods, main text), and the phylogenetic tree of the non-conserved state used. This approximates an estimate of a phylogenetic tree from all the unconstrained sites in the genome.

Di-nucleotide model

The di-nucleotide model is used to define the probability of combined columns of paired nucleotides (main text, fig. 6). It should therefore capture the charac-

teristics of their substitution process, which includes an overall low probability of observing non-pairing di-nucleotides and a low probability of observing substitutions from pairing di-nucleotides to non-pairing di-nucleotides.

We found that the following parameterization achieved this with a reasonable number of free parameters: Let a and b be two di-nucleotides (i.e. states of the process), and let q_{ab}^{di} be the off diagonal entries of the 16x16 rate matrix (Q^{di}). We then define Q^{di} as follows:

$$q_{ab}^{di} = \begin{cases} \pi_b \alpha_{ab} & \text{if both } a \text{ and } b \text{ are pairing} \\ \pi_b \gamma & \text{if either } a \text{ or } b \text{ is pairing} \\ \pi_b \delta & \text{if neither } a \text{ nor } b \text{ is pairing} \end{cases}$$

The set of pairing nucleotides is defined as $\mathcal{A}^{pairing} = \{AT, TA, GC, CG, GT, TG\}$ (T is used instead of U since the input is DNA). α_{ab}, γ, δ define free parameters, which determine the flux (rate of change) between the different pairs of di-nucleotides. The flux between each pair of pairing di-nucleotides is described by its own free parameter

$$\alpha_{ab} = \alpha_{ba} \quad \forall a, b \in \mathcal{A}^{pairing}.$$

There are thus 15 ($6 * 5/2$) such free parameters. The flux between the set of pairing di-nucleotides and the set of non-pairing di-nucleotides is defined by a single parameter (γ), and the flux between any pair of non-pairing di-nucleotides is defined by a single parameter (δ). This model thus has 17 free flux parameters and 15 free equilibrium distribution parameters, for a total of 32 free parameters.

This di-nucleotide model can be seen as a hybrid between a heavily constrained 16-state model and a general reversible six-state model [20]. It is important to use a 16-state model when detecting stem-pairing regions, since the six and seven-state models [20] cannot take changes between non-pairing di-nucleotides into account and will make columns with many such changes too probable.

Single-nucleotide model

The single-nucleotide model is used to define the probability of single unpaired columns both within the loop and bulge regions of the RNA structures and in the surrounding unstructured regions.

Differences in the probability of input columns under this model compared to the di-nucleotide model determines if a structure will be predicted in a given region. It is therefore important that the di-nucleotide model does not favor some regions simply because of, e.g., their base composition, which would lead to spurious structure predictions. On the other hand, it is important that the two models assign significantly different probabilities to truly pairing columns.

We achieve this by defining the substitution process of the single-nucleotide model as an average of the marginal substitution process observed in the left and right positions of the di-nucleotide model. The rate matrix (Q^{single}) and the equilibrium distribution (π^{single}) of the single nucleotide model (ψ^{single}) is thus completely defined in terms of the di-nucleotide model.

The marginalized rate matrix and equilibrium distribution of the single nucleotide model are calculated as follows: Let a, b be states of the single nucleotide substitution process and let c, d be states of the di-nucleotide substitution process. Let c_1 and c_2 denote the left and the right nucleotide of the di-nucleotide c, respectively.The entries of the the equilibrium distribution for the left process are given by $\pi_a^{left} = \sum_{c:c_1=a} \pi_c^{di}$ and correspondingly the entries of the right process are given by $\pi_a^{right} = \sum_{c:c_2=a} \pi_c^{di}$. The rate matrix entries $q_{a,b}$ for the left and the right processes are:

$$
\begin{aligned}
q_{a,b}^{left} &= \sum_{c:c_1=a} \sum_{d:d_1=b} \frac{\pi_c^{di}}{\pi_a^{left}} q_{c,d}^{di} \\
q_{a,b}^{right} &= \sum_{c:c_2=a} \sum_{d:d_2=b} \frac{\pi_c^{di}}{\pi_a^{right}} q_{c,d}^{di}.
\end{aligned}
$$

The final equilibrium distribution and rate matrix of the single nucleotide model are now given by: $\pi^{single} = \frac{1}{2}(\pi^{left} + \pi^{right})$ and $Q^{single} = \frac{1}{2}(Q^{left} + Q^{right})$. This marginalization strategy is inspired by Yang et al. (1998) , who derived an amino-acid substitution process from a codon substitution process.

This scheme thus removes all differences between the models, apart from the ability of the di-nucleotide model to detect correlations between pairing columns. The overall substitution rate is also kept as a free parameter, as explained above (see Phylogenetic tree section).

Algorithms

The algorithms for the phylo-SCFGs are the traditional SCFG algorithms CYK, inside, inside-outside [12, 22, 23]. The only difference is that the emission probabilities are not explicitly defined, but calculated using Felsenstein's algorithm [24], which calculate the probability of an alignment column under a phylogenetic tree.

The SCFG algorithms have cubic running times ($O(L^3)$) in the length of the input sequence, and Felsenstein's algorithm has a linear running time ($O(n)$) in the number of sequences of the alignment. The overall running times of all the phylo-SCFG algorithms are thus $O(L^3 n)$ [11, 13].

Training

Only the fRNA model is trained, since its non-structural component completely specifies the background model.

The state transitions are estimated from known RNA secondary structure annotations, which completely specify the state transitions within the grammar. The EM-algorithm was used to estimate the state transitions, but with no missing data the transition probabilities are in reality estimated by simple counting [12].

The set of pairing columns are also completely specified by the annotation of the training data. A combination of the inside algorithm with Felsenstein's algorithm is used to calculate the likelihood of the training data. The maximum-likelihood estimates of the free parameters of the di-nucleotide substitution model (Q^{di} and π^{di}) are found using the quasi-Newton method with BFGS estimation of the Hessian as implemented in OPT++ [25].

The phylogenetic tree (τ and β) is taken as input (explained above).

Structure prediction

The CYK algorithm (combined with Felsenstein's algorithm) is used to find the most probable parse from the phylo-SCFG. EvoFold returns the corresponding secondary structure as its prediction. The prior (defined by transition probabilities) of the fRNA model is biased toward predictions devoid of structure.

Score calculation

The inside algorithm (combined with Felsenstein's algorithm) is used to calculate the likelihood of the input alignment (x) under both the fRNA model and the background model. The returned folding potential score (FPS) is the log odds between these:

$$FPS = log(P(x|\phi_{fRNA})/P(x|\phi_{bg})).$$

The scores are calculated for specific folds, which are then length normalized.

Position-specific reliability scores

The position-specific reliability scores are given by the posterior probability of observing the assigned annotation. It is calculated using the inside-outside algorithm (combined with Felsenstein's algorithm).

Implementation

The phylo-SCFGs are specified in XML and implemented using the SCFG framework presented in Pedersen et al. (2004) , with specific extensions to deal with rate matrix marginalization, etc.. Post-processing of the outputs (to calculate the FPS from likelihood values, etc.) is done using various python scripts.

Supplementary data

The complete set of predictions can be retrieved in bulk or browsed interactively at the UCSC Human Genome Browser (`http://genome.ucsc.edu/`). The top-ranking folds of each category as well as the set of paralogous families can be accessed from the EvoFold web-site (`http://www.cbse.ucsc.edu/~jsp/EvoFold`). A statically linked linux binary of the EvoFold program can also be downloaded from the EvoFold web-site (source-code is available upon request).

References

[1] Green P, Ewing B, Miller W, Thomas PJ, Green ED (2003) Transcription-associated mutational asymmetry in mammalian evolution. Nat Genet 33: 514–517.

[2] McCutcheon JP, Eddy SR (2003) Computational identification of non-coding RNAs in Saccharomyces cerevisiae by comparative genomics. Nucleic Acids Res 31: 4119–4128.

[3] Babak T, Blencowe BJ, Hughes TR (2005) A systematic search for new mammalian noncoding RNAs indicates little conserved intergenic transcription. BMC Genomics 6: 104.

[4] Griffiths-Jones S (2004) The microRNA Registry. Nucleic Acids Res 32: D109–D111.

[5] Griffiths-Jones S, Moxon S, Marshall M, Khanna A, Eddy SR, et al. (2005) Rfam: annotating non-coding RNAs in complete genomes. Nucleic Acids Res 33: D121–D124.

[6] Lestrade L, Weber MJ (2006) snoRNA-LBME-db, a comprehensive database of human H/ACA and C/D box snoRNAs. Nucleic Acids Res 34: 158–162.

[7] Lowe TM, Eddy SR (1997) tRNAscan-SE: a program for improved detection of transfer RNA genes in genomic sequence. Nucleic Acids Res 25: 955–964.

[8] Lim LP, Glasner ME, Yekta S, Burge CB, Bartel DP (2003) Vertebrate microRNA genes. Science 299: 1540.

[9] Chomsky N (1959) On Certain Formal Properties of Grammars. Information and Control 2: 137–167.

[10] Baker JK (1979) Trainable grammars for speech recognition, in: Speech Communication Papers for the 97th Meeting of the Acoustical Society of America, Boston, MA, pp. 547–550.

[11] Pedersen JS, Meyer IM, Forsberg R, Simmonds P, Hein J (2004) A comparative method for finding and folding RNA secondary structures within protein-coding regions. Nucleic Acids Res 32: 4925–4936.

[12] Durbin R, Eddy S, Krogh A, Mitchison G (1998) Biological sequence analysis: Probabilistic models of proteins and nucleic acids, Cambridge: Cambridge University Press.

[13] Knudsen B, Hein J (1999) RNA Secondary Structure Prediction Using stochastic context-free grammars and evolutionary history. Bioinformatics 15: 446–454.

[14] Jukes TH, Cantor CR (1969) Mammalian Protein Metabolism, New York: Academic Press, chapter 24, pp. 21–132.

[15] Felsenstein J (2003) Inferring Phylogenies., Sinauer Assoc., 664 pp.

[16] Pedersen JS, Hein J (2003) Gene finding with a hidden Markov model of genome structure and evolution. Bioinformatics 19: 219–227.

[17] Boffelli D, McAuliffe J, Ovcharenko D, Lewis KD, Ovcharenko I, et al. (2003) Phylogenetic shadowing of primate sequences to find functional regions of the human genome. Science 299: 1391–1394.

[18] Siepel A, Haussler D (2004) Computational Identification of Evolutionary conserved exons, in: Proceedings of the Eighth Annual International Conference on Computational Biology (RECOMB-04), New York: ACM Press.

[19] Siepel A, Bejerano G, Pedersen JS, Hinrichs AS, Hou M, et al. (2005) Evolutionarily conserved elements in vertebrate, insect, worm, and yeast genomes. Genome Res 15: 1034–1050.

[20] Savill NJ, Hoyle DC, Higgs PG (2001) Rna sequence evolution with secondary structure constraints: comparison of substitution rate models using maximum-likelihood methods. Genetics 157: 399–411.

[21] Yang Z, Nielsen R, Hasegawa M (1998) Models of amino acid substitution and applications to mitochondrial protein evolution. Mol Biol Evol 15: 1600–1611.

[22] Sakakibara Y, Brown M, Underwood R, Mian IS, Haussler D (1994) Stochastic Context-Free Grammars for Modeling RNA, in: Proceedings of the 27th Hawaii International Conference on System Sciences, Honolulu: IEEE Computer Society Press, pp. 284–283.

[23] Eddy SR, Durbin R (1994) RNA sequence analysis using covariance models. Nucleic Acids Res 22: 2079–2088.

[24] Felsenstein J (1981) Evolutionary trees from DNA sequences: a maximum likelihood approach. J Mol Evol 17: 368–376.

[25] Meza JC (1994) OPT++: An Object-Oriented Class Library for Nonlinear Optimization, Technical Report SAND94-8225, Sandia National Laboratories.
