# Supplementary material for: Identification and Classification of Conserved RNA Secondary Structures in the Human Genome
Source: PLoS Comput Biol. 2006 Apr 21;2(4):e33. doi: 10.1371/journal.pcbi.0020033 (PMC1440920; doi:10.1371/journal.pcbi.0020033)
Supplement: Supplementary file 2 [file pcbi.0020033.sg001.pdf]

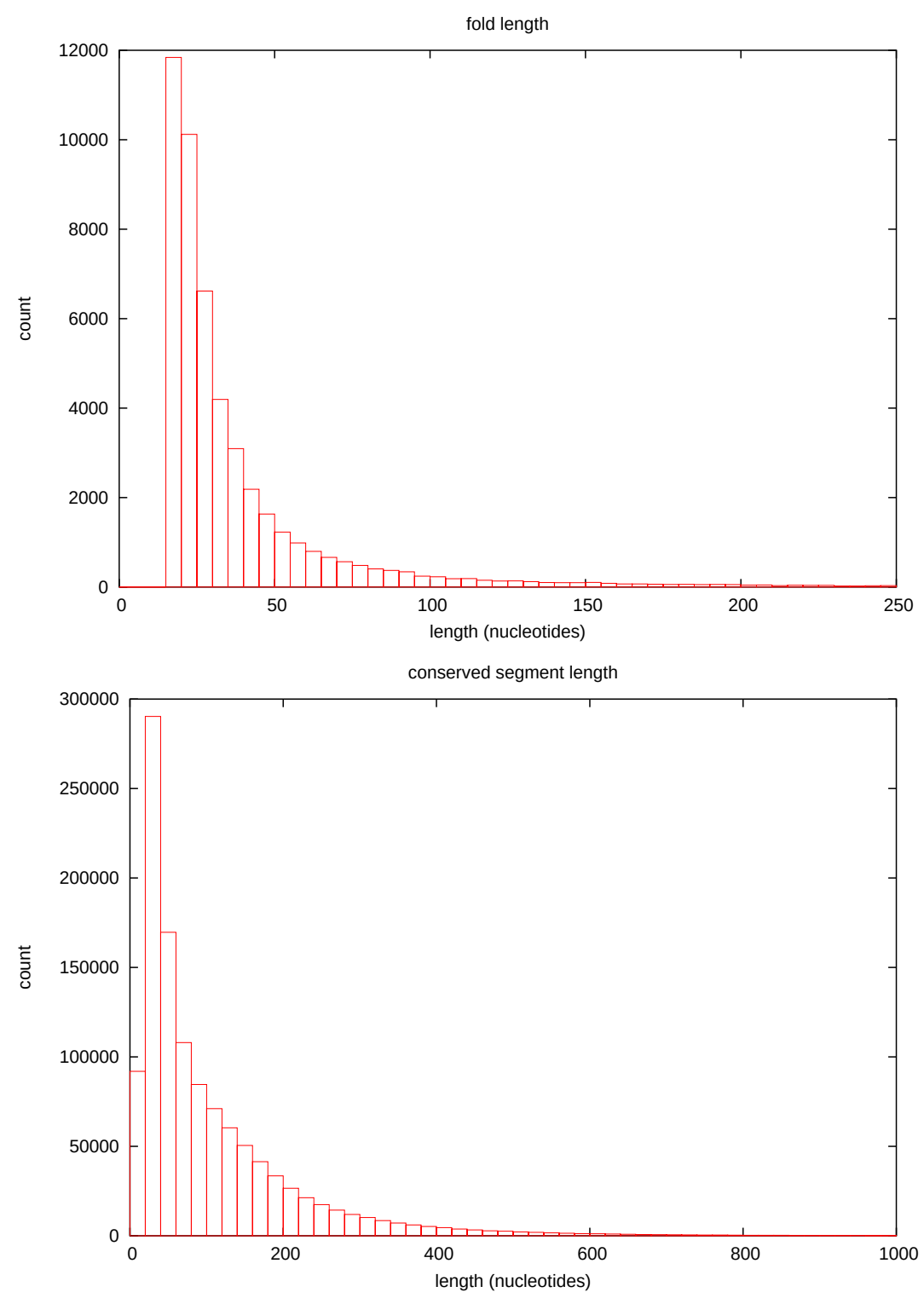

Figure S1: Length of folds (top) and conserved segments (bottom) versus frequency counts. There are 252 folds longer than 250 nucleotides and 1727 conserved segments longer than 1000 nucleotides, which are not included in the above plots.
