# Supplementary material for: Identification and Classification of Conserved RNA Secondary Structures in the Human Genome
Source: PLoS Comput Biol. 2006 Apr 21;2(4):e33. doi: 10.1371/journal.pcbi.0020033 (PMC1440920; doi:10.1371/journal.pcbi.0020033)
Supplement: Supplementary file 3 [file pcbi.0020033.sg002.pdf]

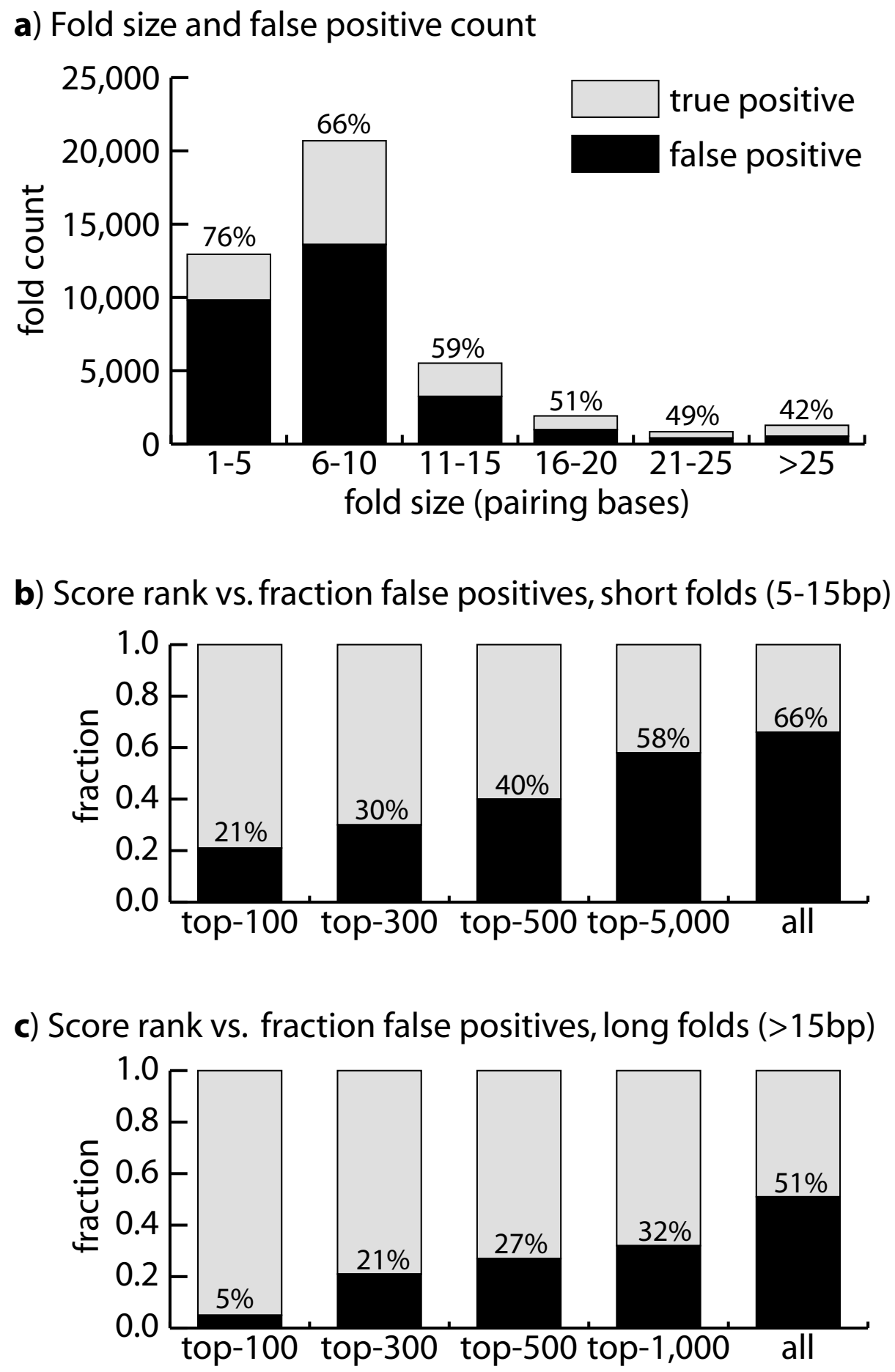


Figure S2: Estimated fraction of false positive predictions. **a**: Count of false positives for different size-ranges of folds. Black bars indicate number of predictions made in randomized alignments (false positives), gray bars indicate the additional number of predictions made in original alignments (true positives). The estimated fraction of false positives is indicated above each column. **b,c**: Fraction of false positives in different top-score-ranked subsets of short folds (fig. b) and long folds (fig. c). Same color coding as for fig. a.
