# Supplementary material for: Identification and Classification of Conserved RNA Secondary Structures in the Human Genome
Source: PLoS Comput Biol. 2006 Apr 21;2(4):e33. doi: 10.1371/journal.pcbi.0020033 (PMC1440920; doi:10.1371/journal.pcbi.0020033)
Supplement: Supplementary file 4 [file pcbi.0020033.sg003.pdf]

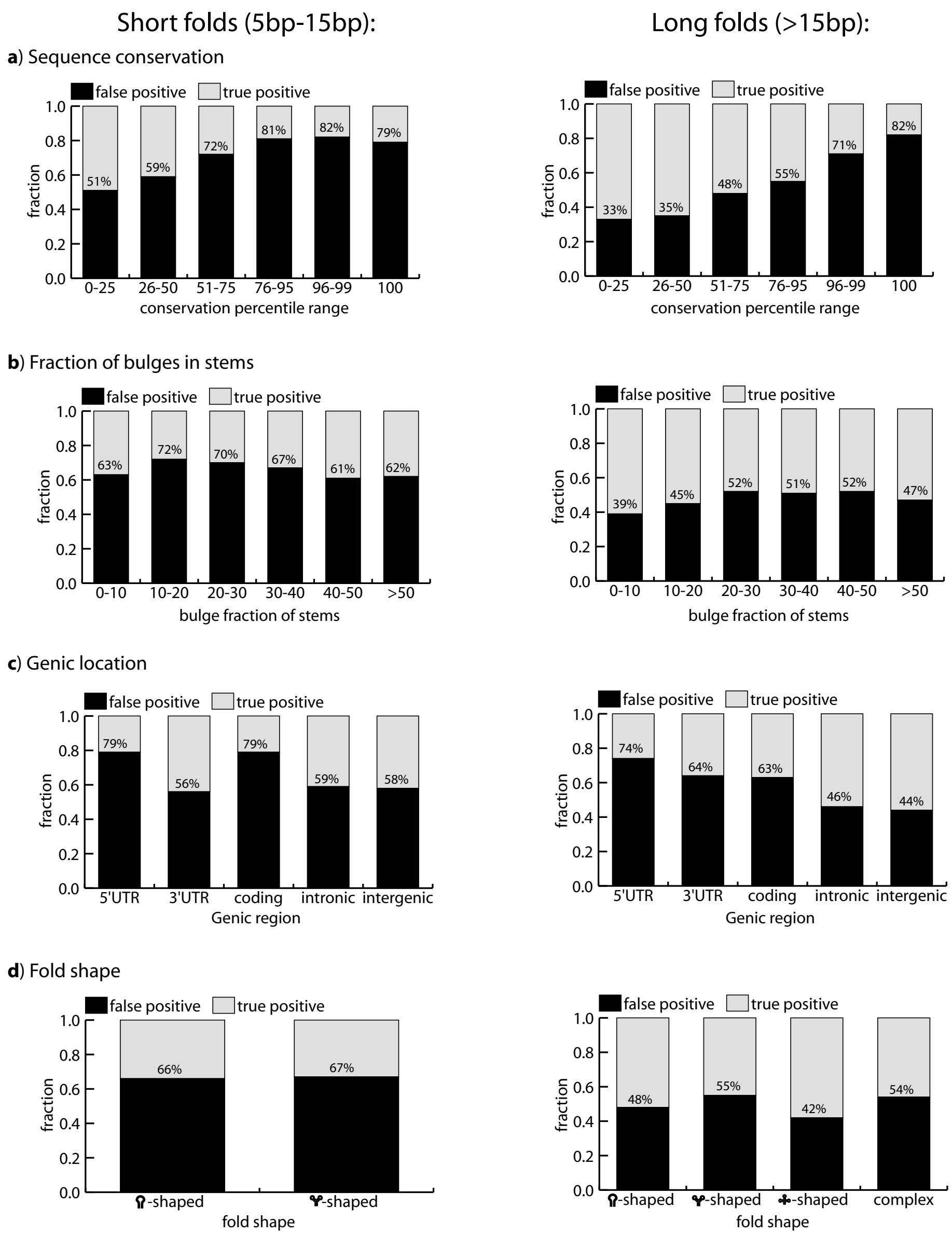

Figure S3: Estimated fraction of false positive predictions as a function of various fold properties for short (left) as well as long (right) folds. For all figures the x-axis gives a measure (or type) of the property in question and the y-axis gives the corresponding fraction of false positive. Definition of properties: **a**: The sequence conservation scores are measured at the input element level and the percentiles are relative to their distribution among all the folds. **b**: The bulge fraction is the percentage of bases in stems found in bulges. **c and d**: The genic location and the fold shape is taken from the fold classification scheme (see methods section of main text for definitions).
