# Supplementary material for: Identification and Classification of Conserved RNA Secondary Structures in the Human Genome
Source: PLoS Comput Biol. 2006 Apr 21;2(4):e33. doi: 10.1371/journal.pcbi.0020033 (PMC1440920; doi:10.1371/journal.pcbi.0020033)
Supplement: Supplementary file 5 [file pcbi.0020033.sg004.pdf]

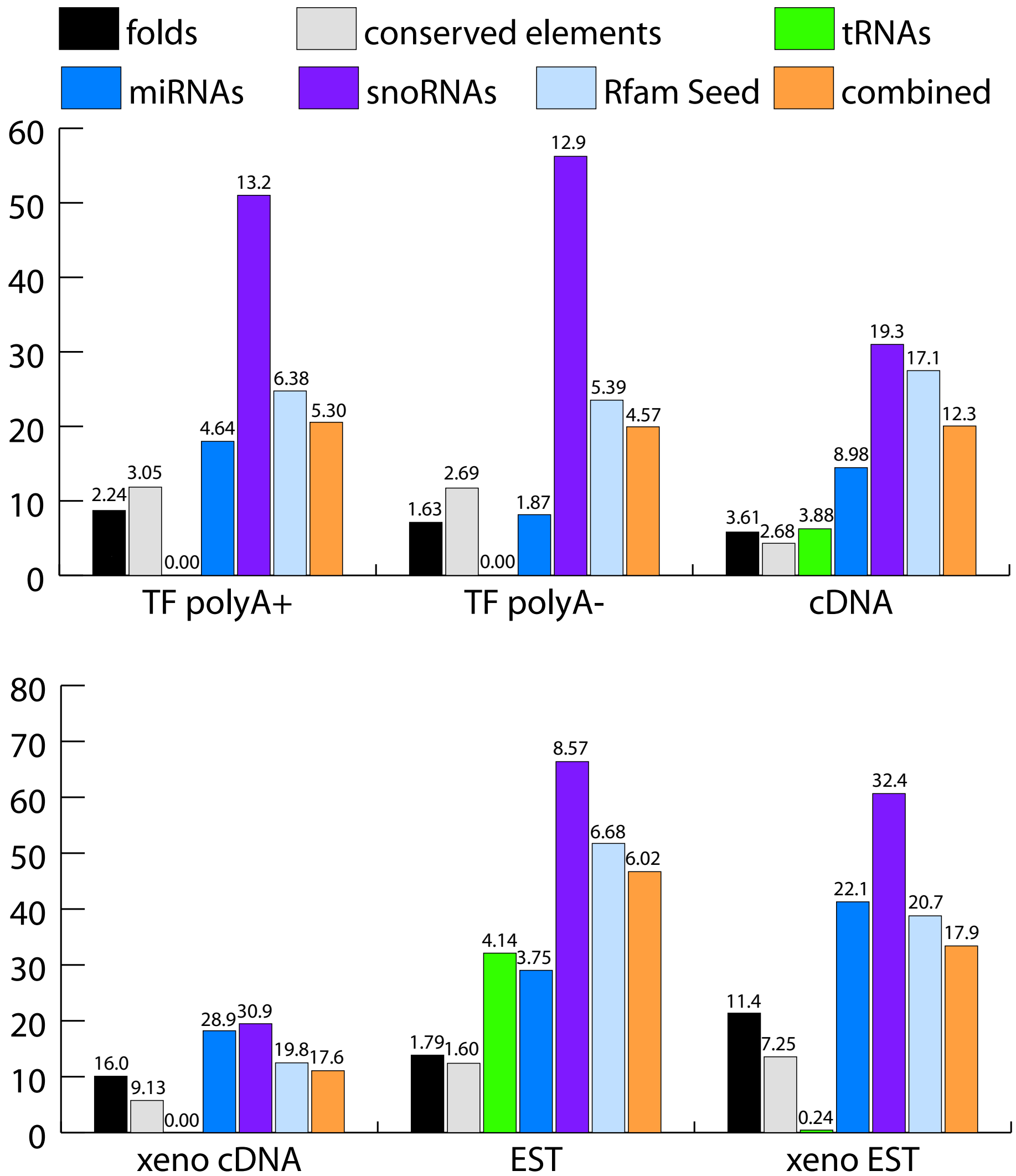

Figure S4: Transcription evidence for predicted folds, conserved elements, and different classes of ncRNAs. The y-axis indicates the coverage in percent. The different types of transcription evidence are given along the x-axis: **TF ployA+**: Transfrags enriched in polyadenylated transcripts, **TF polyA-**: Transfrags depleted of polyadenylated transcripts, **cDNA**: human cDNAs, **xeno cDNA** non-human cDNAs, **EST**: human ESTs, **xeno EST**: non-human ESTs. The enrichment for a given type of transcription evidence relative to the genome-wide coverage of intronic and intergenic regions is given above each column. The combined class combines the tRNAs, miRNAs, snoRNAs, and the Rfam Seed non-coding RNAs.
