# Supplementary material for: Identification and Classification of Conserved RNA Secondary Structures in the Human Genome
Source: PLoS Comput Biol. 2006 Apr 21;2(4):e33. doi: 10.1371/journal.pcbi.0020033 (PMC1440920; doi:10.1371/journal.pcbi.0020033)
Supplement: Supplementary file 6 [file pcbi.0020033.sg005.pdf]

a)

$$
\begin{aligned}
\text{begin\_non-structrual} &\rightarrow \text{unpaired} \\
\text{unpaired} &\rightarrow \text{unpaired } x \mid \text{end } x
\end{aligned}
$$

b)

$$
\begin{aligned}
\text{begin\_structrual} &\rightarrow \text{stem\_pair} \\
\text{stem\_pair} &\rightarrow x_l \text{ stem\_pair } x_r \mid x_l \text{ bifurcation } x_r \\
\text{bifurcation} &\rightarrow \text{emit intermediate} \\
\text{emit} &\rightarrow \text{loop\_\&\_bulge } \mid \text{stem\_pair} \\
\text{loop\_\&\_bulge} &\rightarrow \text{end } x \\
\text{intermediate} &\rightarrow \text{bifurcation} \mid \text{emit}
\end{aligned}
$$

Figure S5: Production rules of the non-structural component (**a**) and the structural component (**b**). Nomenclature: '|' denotes a choice between different productions; x denotes single column emissions; x_l and x_r denotes the left and right part of pair emissions, respectively. A corresponding graphical overview of these grammar components are given in fig. S6.
