# Supplementary material for: Identification and Classification of Conserved RNA Secondary Structures in the Human Genome
Source: PLoS Comput Biol. 2006 Apr 21;2(4):e33. doi: 10.1371/journal.pcbi.0020033 (PMC1440920; doi:10.1371/journal.pcbi.0020033)
Supplement: Supplementary file 7 [file pcbi.0020033.sg006.pdf]

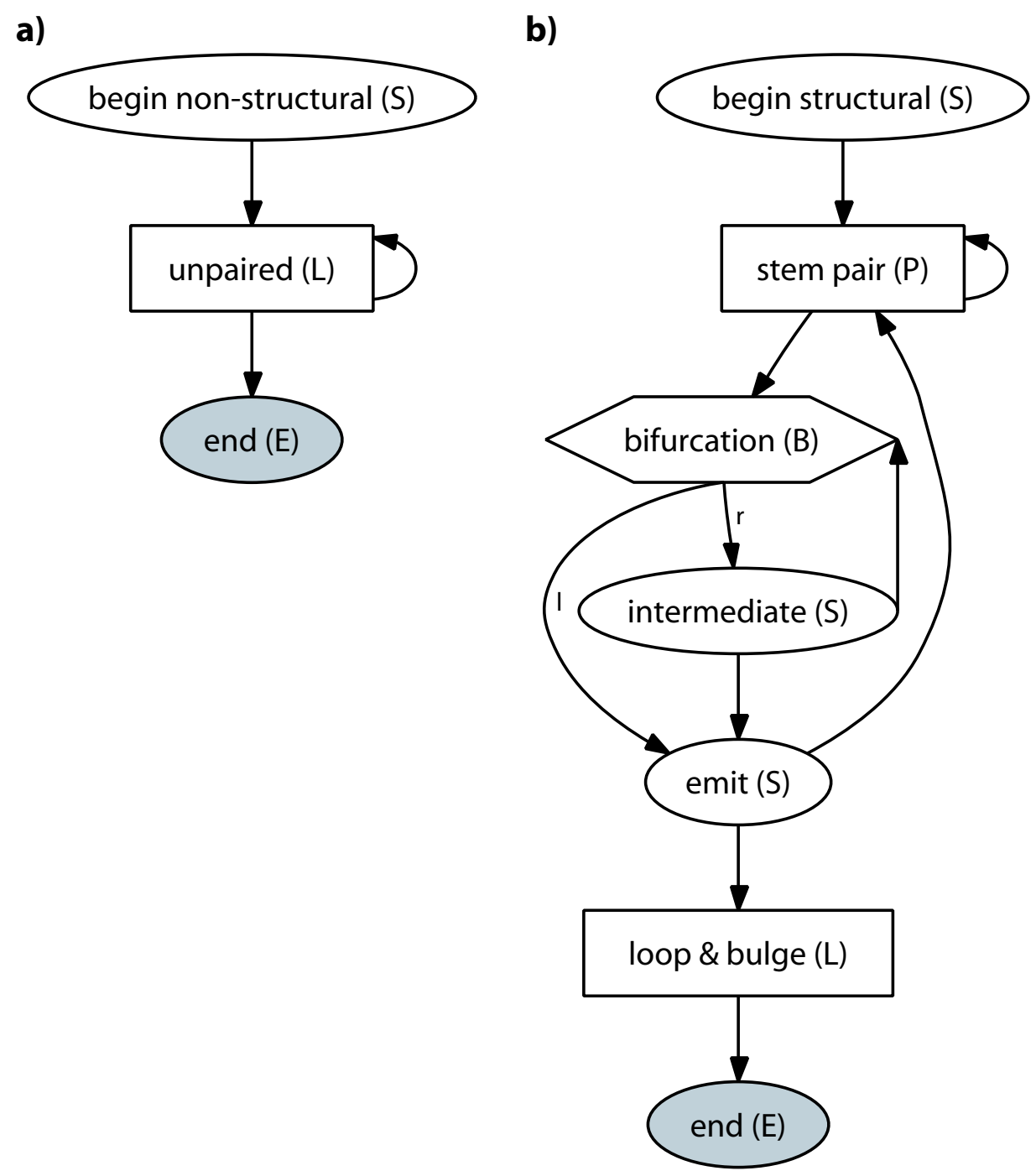

Figure S6: Transition graphs of the non-structural component (**a**) and the structural component (**b**) of the phylo-SCFGs. The state types are given in parenthesis. Arrows indicate possible state transitions. The transition from the *bifurcation* state leads to two states, a left (l) and a right (r), as indicated on the graph. The *unpaired* and the *loop & bulge* states have associated single-column emission distributions (specified by a single-nucleotide phylogenetic model). The *stem pair* state has an associated di-column emission distribution (specified by a di-nucleotide phylogenetic model).
