# Supplementary material for: Identification and Classification of Conserved RNA Secondary Structures in the Human Genome
Source: PLoS Comput Biol. 2006 Apr 21;2(4):e33. doi: 10.1371/journal.pcbi.0020033 (PMC1440920; doi:10.1371/journal.pcbi.0020033)
Supplement: Supplementary file 8 [file pcbi.0020033.st001.pdf]

Table S2: Count statistics for short fold classes

type\location	5'UTR			3'UTR			coding			intronic			intergenic			any location		
hairpin	263	(20%)	53	2567	(44%)	1139	10945	(20%)	2217	8965	(41%)	3668	15667	(42%)	6538	38407	(35%)	13614
Y-shaped	3	(67%)	2	49	(11%)	5	327	(30%)	99	120	(50%)	60	160	(33%)	52	659	(33%)	219
clover-shaped	0	(n.a.)	0	0	(n.a.)	0	6	(80%)	5	1	(0%)	0	1	(100%)	1	8	(75%)	6
complex shapes	0	(n.a.)	0	0	(n.a.)	0	0	(n.a.)	0	1	(0%)	0	0	(n.a.)	0	1	(0%)	0
any shape	266	(21%)	55	2616	(44%)	1144	11278	(21%)	2321	9087	(41%)	3728	15828	(42%)	6591	39075	(35%)	13839

The fold counts, estimated true positive rate (in parenthesis), and estimated true positive counts are given for each location/shape-class of short folds. The "any shape" row and the "any location" column gives the marginalized counts for each set of fold classes. The entry at the lower right corner thus holds the overall counts for the set of long folds.
