# Supplementary material for: Identification and Classification of Conserved RNA Secondary Structures in the Human Genome
Source: PLoS Comput Biol. 2006 Apr 21;2(4):e33. doi: 10.1371/journal.pcbi.0020033 (PMC1440920; doi:10.1371/journal.pcbi.0020033)
Supplement: Supplementary file 9 [file pcbi.0020033.st002.pdf]

Table S3: Count statistics for long fold classes

type\location	5'UTR			3'UTR			coding			intronic			intergenic			any location		
hairpin	32	(56%)	18	359	(37%)	132	666	(36%)	237	1262	(56%)	702	2238	(59%)	1325	4557	(53%)	2413
Y-shaped	20	(0%)	0	205	(36%)	74	548	(39%)	213	803	(49%)	397	1244	(50%)	623	2820	(46%)	1307
clover-shaped	4	(0%)	0	17	(100%)	17	39	(37%)	14	71	(61%)	43	111	(69%)	77	242	(63%)	152
complex shapes	12	(33%)	4	134	(29%)	39	205	(39%)	81	554	(55%)	307	880	(46%)	402	1785	(47%)	833
any shape	68	(32%)	22	715	(37%)	261	1458	(37%)	546	2690	(54%)	1449	4473	(54%)	2427	9404	(50%)	4705

See legend of Table S2.
