# Supplementary material for: Identification and Classification of Conserved RNA Secondary Structures in the Human Genome
Source: PLoS Comput Biol. 2006 Apr 21;2(4):e33. doi: 10.1371/journal.pcbi.0020033 (PMC1440920; doi:10.1371/journal.pcbi.0020033)
Supplement: Supplementary file 10 [file pcbi.0020033.st003.pdf]

Table S1: Strand bias of EvoFold predictions

Genic region	count	ass. statistic	P-value [b]
coding	10551	0.496	0.47
5'UTR	207	0.553	0.164
3'UTR	2725	0.646	$< 2.2e-16$
intron	9603	0.558	$< 2.2e-16$
combined	23086	0.549	$< 2.2e-16$

[b] The association statistic was assumed to be binomial distributed with parameter p=0.5. The alternative hypothesis is that p deviates from 0.5.
