# Supplementary material for: Identification and Classification of Conserved RNA Secondary Structures in the Human Genome
Source: PLoS Comput Biol. 2006 Apr 21;2(4):e33. doi: 10.1371/journal.pcbi.0020033 (PMC1440920; doi:10.1371/journal.pcbi.0020033)
Supplement: Supplementary file 11 [file pcbi.0020033.st004.pdf]

Table S4: EvoFold sensitivity using only human and mouse sequences

Data set	sensitivity		relative sensitivity	
miRNA registry [1]	48%	(88/183)	56%	(88/157)
Histone 3'UTR stem-loops [2]	0%	(0/64)	0%	(0/62)
snoRNAs [6]	2%	(4/190)	40%	(4/10)
tRNAs [3]	0%	(0/2)	0%	(0/2)
Rfam Seed [2]	18%	(41/231)	41%	(41/100)

The sensitivity column gives the number of known fRNAs recognized by EvoFold using the human-mouse sub-alignment divided by the total number of fRNAs in the input segments. The relative sensitivity column gives the ratio between the sensitivity using only the human and mouse sub-alignment and the complete 8-way alignment.

References:

[1] Griffiths-Jones S (2004) The microRNA Registry. Nucleic Acids Res 32: D109–D111.

[2] Griffiths-Jones S, Moxon S, Marshall M, Khanna A, Eddy SR, et al. (2005) Rfam: annotating non-coding RNAs in complete genomes. Nucleic Acids Res 33: D121–D124.

[3] Lestrade L, Weber MJ (2006) snoRNA-LBME-db, a comprehensive database of human H/ACA and C/D box snoRNAs. Nucleic Acids Res 34: 158–162.

[4] Lowe TM, Eddy SR (1997) tRNAscan-SE: a program for improved detection of transfer RNA genes in genomic sequence. Nucleic Acids Res 25: 955–964.
